# Aflibercept exhibits VEGF binding stoichiometry distinct from bevacizumab and does not support formation of immune-like complexes

**DOI:** 10.1007/s10456-016-9515-8

**Published:** 2016-05-27

**Authors:** Douglas A. MacDonald, Joel Martin, Kathir K. Muthusamy, Jiann-Kae Luo, Erica Pyles, Ashique Rafique, Tammy Huang, Terra Potocky, Yang Liu, Jingtai Cao, Françoise Bono, Nathalie Delesque, Pierre Savi, John Francis, Ali Amirkhosravi, Todd Meyer, Carmelo Romano, Meredith Glinka, George D. Yancopoulos, Neil Stahl, Stanley J. Wiegand, Nicholas Papadopoulos

**Affiliations:** Regeneron Pharmaceuticals Inc., 777 Old Saw Mill River Road, Tarrytown, NY 10591 USA; Sanofi, Toulouse, France; Center for Thrombosis Research, Florida Hospital, Orlando, FL USA

**Keywords:** VEGF, Bevacizumab, Aflibercept, Immune complexes

## Abstract

Anti-vascular endothelial growth factor (VEGF) therapies have improved clinical outcomes for patients with cancers and retinal vascular diseases. Three anti-VEGF agents, pegaptanib, ranibizumab, and aflibercept, are approved for ophthalmic indications, while bevacizumab is approved to treat colorectal, lung, and renal cancers, but is also used off-label to treat ocular vascular diseases. The efficacy of bevacizumab relative to ranibizumab in treating neovascular age-related macular degeneration has been assessed in several trials. However, questions persist regarding its safety, as bevacizumab can form large complexes with dimeric VEGF_165_, resulting in multimerization of the Fc domain and platelet activation. Here, we compare binding stoichiometry, Fcγ receptor affinity, platelet activation, and binding to epithelial and endothelial cells in vitro for bevacizumab and aflibercept, in the absence or presence of VEGF. In contrast to bevacizumab, aflibercept forms a homogenous 1:1 complex with each VEGF dimer. Unlike multimeric bevacizumab:VEGF complexes, the monomeric aflibercept:VEGF complex does not exhibit increased affinity for low-affinity Fcγ receptors, does not activate platelets, nor does it bind to the surface of epithelial or endothelial cells to a greater degree than unbound aflibercept or control Fc. The latter finding reflects the fact that aflibercept binds VEGF in a unique manner, distinct from antibodies not only blocking the amino acids necessary for VEGFR1/R2 binding but also occluding the heparin-binding site on VEGF_165_.

**Electronic supplementary material:**

The online version of this article (doi:10.1007/s10456-016-9515-8) contains supplementary material, which is available to authorized users.

## Introduction

Angiogenesis, the growth of new blood vessels from preexisting vasculature, is a highly orchestrated process that is critical for proper embryonic and postnatal vascular development [1]. Abnormal or pathological angiogenesis is a hallmark of cancer and several retinal diseases where the upregulation of proangiogenic factors, such as vascular endothelial growth factor (VEGF) and placental growth factor (PlGF), leads to increases in endothelial proliferation, changes in vasculature morphology, and increased vascular permeability [2, 3]. In particular, blockade of VEGF has shown clinical utility in the oncology setting as well as several retinal vascular diseases characterized by abnormal angiogenesis and/or vascular permeability, such as the “wet” form of age-related macular degeneration (AMD), the leading cause of blindness in the elderly [4, 5]. The formation of new blood vessels and vascular leakage in wet AMD leads to macular edema, thickening of the retina, and loss of vision. Elevated levels of VEGF have been found in the vitreous fluid and retinal vasculature of patients with AMD [6]. Blocking VEGF activity has also become the therapy of choice for treating diabetic macular edema (DME), retinal vein occlusions, and other ocular diseases where abnormal angiogenesis is the underlying etiology [7–10].

Several anti-VEGF therapies have been approved for use in neovascular or “wet” AMD. Pegaptanib, an oligonucleotide aptamer that binds the heparin-binding domain of VEGF_165_, was the first anti-VEGF therapy that showed some benefit in treating wet AMD, although most patients still experienced visual decline [11]. The introduction of ranibizumab, an affinity-matured, humanized monoclonal antibody fragment (Fab), represented a major advance in treating wet AMD patients since it was not only able to stabilize but in many cases improve visual acuity [12]. However, prior to regulatory approval of ranibizumab, ophthalmologists began using bevacizumab, a humanized monoclonal antibody to VEGF already approved for metastatic colorectal cancer, to treat wet AMD. Like ranibizumab, bevacizumab binds to all isoforms of VEGF-A, but is aliquoted and repackaged for off-label use in ophthalmological indications [13]. Several, small, non-randomized studies evaluated bevacizumab as a potential treatment option in the treatment for wet AMD and thus began the ongoing debate on the merits of utilizing bevacizumab for the treatment for diseases affecting the ocular vasculature [14]. More recently, in a large (*n* = 1208), prospective, randomized comparison of age-related macular degeneration treatments trial (CATT), comparison of the same regimens of ranibizumab or bevacizumab demonstrated similar improvements for the primary end point of visual acuity at 1 year [15]. However, somewhat higher rates of systemic serious adverse events (SAEs) were observed in patients treated with bevacizumab compared to ranibizumab. Curiously, the excess in systemic SAEs observed in bevacizumab-treated patients in the CATT trial did not correspond to adverse cardiovascular events, such as hypertension and arteriothrombolic events (ATEs), previously known to be caused by systemic VEGF inhibition in oncology trials that employed much higher, intravenous doses of bevacizumab. Rather, patients treated intravitreally with low-dose bevacizumab exhibited increases in SAEs affecting other organ systems, particularly gastrointestinal disorders. While two additional, but smaller trials (IVAN; *n* = 610, MANTA; *n* = 321) individually showed no statistically significant differences in systemic SAEs between ranibizumab and bevacizumab [16, 17], a recent meta-analysis (*n* = 3665) comprising the above studies as well as six additional trials also found a higher incidence of gastrointestinal disorders in patients treated with intravitreal bevacizumab compared to ranibizumab, with no differences in other systemic SAEs [18]. In comparing the safety profile of ranibizumab to bevacizumab when treating DME, the recent Protocol T trial showed no differences in the rates of serious adverse events among all three anti-VEGF treatments (aflibercept, bevacizumab, ranibizumab), though visual acuity gains with aflibercept were significantly greater than with ranibizumab or bevacizumab in the overall population, and these differences were especially evident in patients with poor vision at the start of treatment [19].

Although mechanisms underlying the potentially higher rate of systemic SAEs not typically associated with anti-VEGF activity in patients receiving intravitreal bevacizumab remains unknown, a number of investigators have pointed out several fundamental differences between ranibizumab and bevacizumab. First, ranibizumab is a Fab fragment and is significantly smaller in size than a full-length antibody. This was advantageous for the development for intravitreal injection as the smaller size of ranibizumab was expected to enhance diffusion from the vitreous into the retina and choroid [20]. VEGF and closely related molecules are ligands for a family of related receptor tyrosine kinases (VEGFRs), and as such exist naturally as dimers. Since ranibizumab is a Fab fragment, two molecules of ranibizumab are bound by each VEGF dimer. In contrast, bevacizumab is an IgG comprising two Fabs as well as an Fc domain. Due to its bivalent nature, a single bevacizumab molecule can bind both active sites in a single VEGF dimer. Interestingly, it has also been reported that bevacizumab has the capacity to form large multimeric complexes with VEGF [21, 22]. Second, though derived from bevacizumab, ranibizumab has been affinity-matured with a reported affinity enhancement of 10- to 100-fold relative to the Fab, which is associated with a similarly enhanced activity of ranibizumab in cell-based assays, compared to the Fab fragment of bevacizumab [23, 24]. However, the bivalent nature of the full-length bevacizumab antibody contributes substantially to its ability to bind and neutralize VEGF. Specifically, avidity interactions of bevacizumab with its dimeric VEGF target significantly increase binding affinity and potency of the bivalent antibody relative to the monovalent antigen-binding fragment [25]. Lastly, ranibizumab does not contain a fragment crystallizable (Fc) region, unlike full-length antibodies. While the Fc moiety is useful to increase circulatory half-life when an antibody is given systemically, via its interactions with the neonatal receptor, the Fc region can also promote effector function if the antibody is multimerized upon binding to its target. In the absence of this multimerization, the Fc domain of therapeutic antibodies rarely occupies Fcγ receptors due to the constant competition with high levels of endogenous IgG (5–15 g/L) [26]. Meyer et al. [22] have shown that bevacizumab and equimolar amounts of VEGF_165_ can form immune-like complexes, which in the presence of heparin can engage the FcγRIIa receptor on thrombocytes leading to platelet activation. Other studies have also reported differences in the activities of ranibizumab and bevacizumab that appear to be unrelated to VEGF neutralization per se. For example, bevacizumab exhibits enhanced binding to and accumulation in retinal pigment epithelium (RPE) cells in vitro, relative to ranibizumab [27].

Aflibercept is a novel soluble decoy receptor consisting of an all-human amino acid sequence comprising the second Ig domain of human VEGFR1 and the third Ig domain of human VEGFR2 expressed as an inline fusion with the constant region (Fc) of human IgG1 [28]. Like bevacizumab and ranibizumab, aflibercept binds all forms of VEGF-A (VEGF) but in addition binds PlGF and VEGF-B [25]. Aflibercept was recently approved for the treatment for wet AMD based on the VIEW studies [29]. These trials demonstrated equivalent improvements in visual acuity at 1 year between patients treated monthly with ranibizumab and those treated with aflibercept every other month following three initial monthly loading doses. Each aflibercept molecule, like bevacizumab, contains two, independent VEGF binding arms held together via the Fc moiety. Upon binding VEGF dimers, aflibercept could in theory form large, multimeric immune-like complexes similar to those previously described for bevacizumab, which in turn could trigger processes mediated by low-affinity Fc receptors.

In the present study, we compare the binding stoichiometries of bevacizumab and aflibercept to VEGF_165_, over a range of relative molar concentrations, and also assessed the binding affinities of bevacizumab and aflibercept to VEGF_121_, VEGF_165_, and Fcγ receptors, alone or when complexed with VEGF. The ability of bevacizumab:VEGF and aflibercept:VEGF complexes to activate human platelets in vitro, and the propensity of both bevacizumab and aflibercept to bind to cultured human retinal epithelial (ARPE-19) and human umbilical vein endothelial cells (HUVEC) in the presence and absence of VEGF_165_ and VEGF_121_ also were assessed. The results of these experiments confirm that bevacizumab can form large multimeric complexes with VEGF, when both antibody and ligand are present in roughly (within tenfold) equimolar concentrations. In contrast, aflibercept forms a homogenous 1:1 complex with VEGFs over all molar ratios tested. Moreover, in contrast to the large and heterogenous bevacizumab:VEGF_165_ complexes, these discrete aflibercept:VEGF_165_ complexes do not activate platelets in the presence of heparin. Finally, aflibercept does not bind to the surface of HUVEC or ARPE-19 cells to an appreciably greater degree than the control, human Fc (hFc), in the presence or absence of VEGFs. In contrast, binding of bevacizumab to the surface of these cells was greatly enhanced in the presence of exogenous or endogenous VEGF_165_, but not VEGF_121_. This finding suggested that the cell surface binding of bevacizumab:VEGF_165_ complexes was mediated by the heparin and/or neuropilin-1 (NRP1)-binding domains of this VEGF isoform. This was confirmed by subsequent surface plasmon resonance (Biacore) experiments, which showed that bevacizumab:VEGF_165_ complexes, but not aflibercept:VEGF_165_ complexes, were readily bound by surface-captured NRP1 or heparin. In conclusion, under certain conditions, bevacizumab can form large, multimeric immune-like complexes that exhibit enhanced binding not only to a range of Fcγ receptors, but also, when complexed with VEGF_165_, to heparin and neuropilin present on the surfaces of cells. In contrast, aflibercept exclusively forms a 1:1 complex with VEGF dimers, which does not appreciably increase binding to low-affinity Fc receptors, heparin, or neuropilin—compared to unbound aflibercept or control, monomeric IgGs. The fundamental difference between bevacizumab and aflibercept behavior appears to be attributable to the unique way in which the aflibercept encompasses or “Traps” a single, VEGF dimer, not only blocking the amino acids necessary for VEGFR1/R2 binding but also occluding the heparin-binding site on VEGF as well.

## Results

### In contrast to bevacizumab, aflibercept forms a 1:1 complex with VEGF

**Fig. 1:**
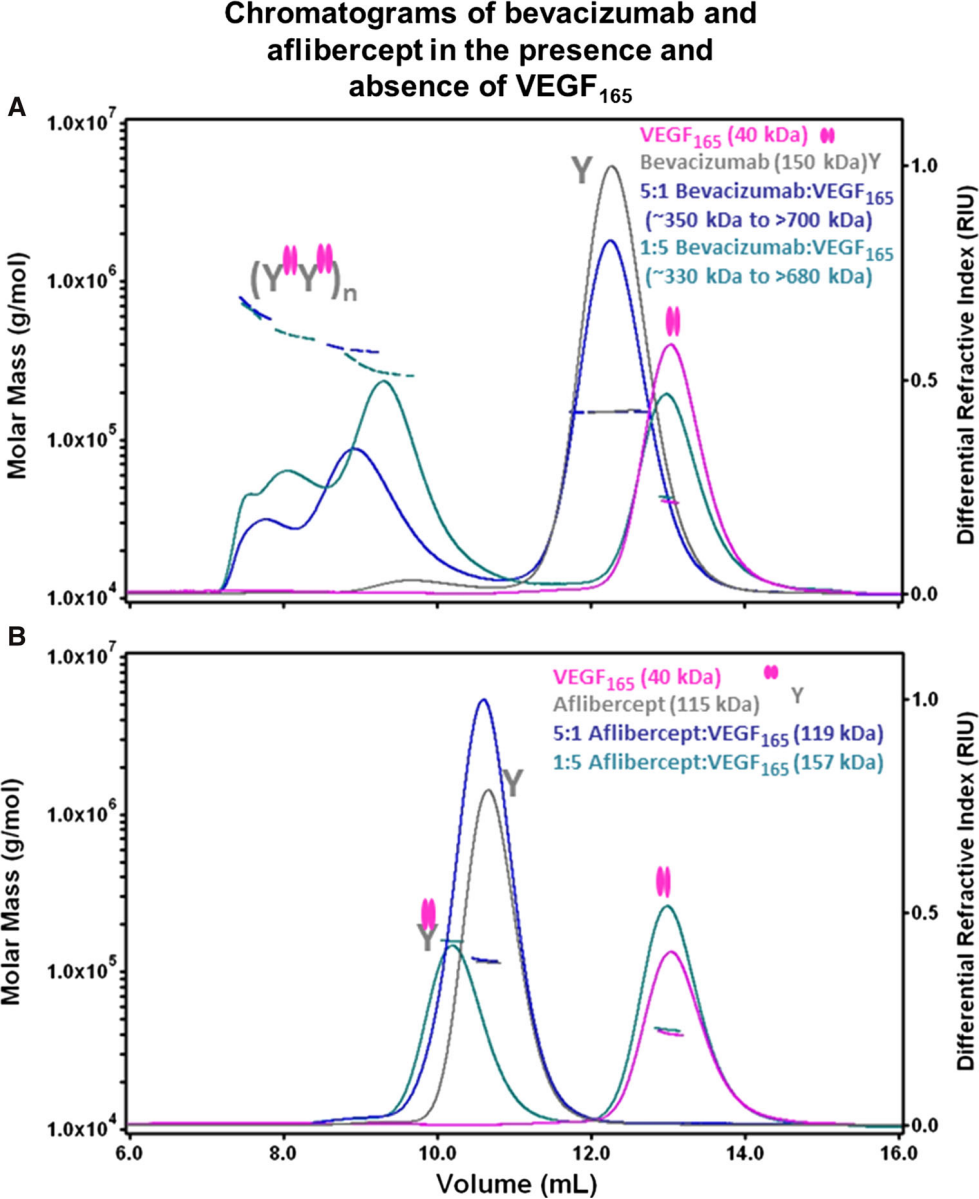
Aflibercept forms 1:1 complexes with VEGF_165_. The molar masses of aflibercept:VEGF_165_ and bevacizumab:VEGF_165_ complexes were analyzed by multi-angle laser light scattering detection coupled to SEC. The differential refractive index (*right* *y* axis) and the measured molar mass (*left* *y* axis) of peaks are indicated as a function of elution volume for each sample. The experimentally determined molar masses are indicated by horizontal lines. Cartoons of free VEGF_165_ and complexes of aflibercept or bevacizumab bound to VEGF_165_ are shown. Complexes of VEGF_165_ with bevacizumab (**a**) or aflibercept (**b**) at various molar ratios were incubated for 12 h at ambient temperature. Following incubation, the samples were kept at 4 °C in the autosampler prior to injection (~100–200 µg per sample) onto a Superose 12 column pre-equilibrated in 10 mM phosphate containing 500 mM NaCl buffer (pH 7.0) with a flow rate of 0.3 mL/min. Chromatograms of VEGF_165_ and bevacizumab (**a**) or aflibercept (**b**) are superimposed to indicate the elution profiles of the unbound proteins. The 1:1 molar ratio complexes yielded similar elution profiles and are not shown for the purposes of clarity

Multiple-angled laser light scattering (MALLS) coupled to size exclusion chromatography (SEC) was used to calculate the stoichiometry of bevacizumab:VEGF_165_ and aflibercept:VEGF_165_ complexes. VEGF_165_ was mixed with either bevacizumab or aflibercept in solution at molar ratios of inhibitor to ligand of between 5:1 and 1:5. Bevacizumab was found to form a heterogeneous mixture of multimeric complexes in the presence of VEGF_165_, with molar masses ranging from ~330 to 700 kDa (Fig. 1a). In contrast, aflibercept at a 1:5 molar ratio showed two discrete peaks (Fig. 1b), as expected based on previous results [21]. The earlier eluting peak, with an apparent molar mass of 157 kDa, corresponded to a 1:1 complex between aflibercept (~115 kDa) and VEGF_165_ (~40 kDa). The latter eluting peak corresponded to the expected excess of free VEGF_165_ dimer (based on the average molar mass of the peak and comparison with the elution times from VEGF_165_ injections). Analysis of a preformed aflibercept:VEGF_165_ complex at a 5:1 molar ratio showed a single peak of 119 kDa (Fig. 1b), which is attributed to incomplete separation between the 1:1 aflibercept:VEGF_165_ complex (~157 kDa) and the excess free aflibercept (~115 kDa).

SEC–MALLS analysis was also used to estimate the binding stoichiometry and molar mass of the complexes formed between bevacizumab or aflibercept and PlGF-2 (Figure S1). No complex formation was observed between bevacizumab and PlGF-2 (Figure S1A). The aflibercept:PlGF-2 complex demonstrated a very similar stoichiometry to the aflibercept:VEGF_165_ complex, with a single major homogenous peak (molar mass of 150 kDa) corresponding to a 1:1 complex between aflibercept and PlGF-2 (~42 kDa) and a minor peak corresponding to an excess of free PlGF-2 dimer (Figure S1B).

### Aflibercept’s binding half-life to Fcγ receptors does not change in the presence of VEGF

Surface plasmon resonance was used to determine the dissociation rate constants (*k*_d_) of bevacizumab and aflibercept to the family of human Fcγ receptors in the presence or absence of VEGF_165_ or VEGF_121_ (Table 1). Equilibrium binding constants (*K*_D_ = *k*_d_/*k*_a_) were not calculated since the heterogenous nature of the bevacizumab:VEGF complex did not allow accurate molar concentrations to be utilized and thus association rate constants (*k*_a_), which are dependent on concentration, could not be determined. Bevacizumab and aflibercept, in the absence of VEGF, exhibited the expected rapid dissociation rate constant (*k*_d_) from low-affinity Fc receptors, with *t*_1/2_ values typical for unbound endogenous IgG1 and recombinant molecules containing IgG1-Fc (*K*_D_ ≈ 1–10 μM) [30]. However, when bevacizumab was pre-incubated in a 1:1 molar ratio with VEGF_165_ or VEGF_121_, the resulting complexes exhibited longer *t*_1/2_ values (slower dissociation rate constants) when bound to the low-affinity Fcγ receptors FcγRIIa, FcγRIIb, FcγRIIIa (176F), FcγRIIIa (176 V), and FcγRIIIb compared to bevacizumab alone. This increase in binding half-life ranged from a ninefold increase when the bevacizumab:VEGF complex was flowed over FcγRIIIa (176 V) to a 177-fold increase in *t*_1/2_ when flowed over FcγRIIb. In contrast, aflibercept with or without VEGF present exhibited no change in *t*_1/2_ values when bound to Fcγ receptors. Representative sensorgrams of bevacizumab and aflibercept binding to Fcγ receptors are shown in Figures S2, S3, S4.

**Table 1:** In vitro binding affinity of bevacizumab and aflibercept for Fcγ receptors in the presence and absence of VEGF_121_ and VEGF_165_ determined by SPR (Biacore)

VEGF inhibitors (1 μM)	Ligand injected (1 μM)	FcγRI			FcγRIIa^b^			FcγRIIb		
		*k*_d_ (1/s)	*t*_1/2_ (s)	*t*_1/2_ Fold changes^a^	*k*_d_ (1/s)	*t*_1/2_ (s)	*t*_1/2_ Fold changes^a^	*k*_d_ (1/s)	*t*_1/2_ (s)	*t*_1/2_ fold changes^a^
Bevacizumab	–	7.49E−04	925	1	1.11E−01	6	1	1.79E−01	4	1
	VEGF_165_	2.41E−04	2874	3	1.02E−03	681	114	9.80E−04	707	177
	VEGF_121_	2.03E−04	3419	4	1.42E−03	487	81	1.82E−03	380	95
Aflibercept	–	7.21E−04	961	1	2.24E−01	3	1	2.55E−01	3	1
	VEGF_165_	6.17E−04	1123	1	2.05E−01	3	1	1.74E−01	4	1
	VEGF_121_	6.83E−04	1015	1	1.86E−01	4	1	2.05E−01	3	1

VEGF inhibitors (1 μM)	Ligand injected (1 μM)	FcγRIIIa (176 V)^c^			FcγRIIIa (176F)^d^			FcγRIIIb		
		*k*_d_ (1/s)	*t*_1/2_ (s)	*t*_1/2_ fold changes^a^	*k*_d_ (1/s)	*t*_1/2_ (s)	*t*_1/2_ fold changes^a^	*k*_d_ (1/s)	*t*_1/2_ (s)	*t*_1/2_ fold changes^a^
Bevacizumab	–	8.03E−03	86	1	6.26E−02	11	1	1.96E−01	4	1
	VEGF_165_	7.78E−04	891	10	1.30E−03	533	48	1.50E−03	463	116
	VEGF_121_	9.09E−04	762	9	1.83E−03	378	34	2.61E−03	265	66
Aflibercept	–	7.62E−03	91	1	4.89E−02	14	1	1.71E−01	4	1
	VEGF_165_	6.14E−03	113	1	3.92E−02	18	1	1.64E−01	4	1
	VEGF_121_	5.73E−03	121	1	3.61E−02	19	1	1.42E−01	5	1

^a^Fold = *t*_1/2_ (ligand + VEGF inhibitor complex)/*t*_1/2_ (VEGF inhibitor alone)

^b^FcγRIIa allele studied was 131R

^c^FcγRIIIa allele studied was 176V

^d^FcγRIIIa allele studied was 176F

### Aflibercept:VEGF_165_ complexes do not activate platelets

**Fig. 2:**
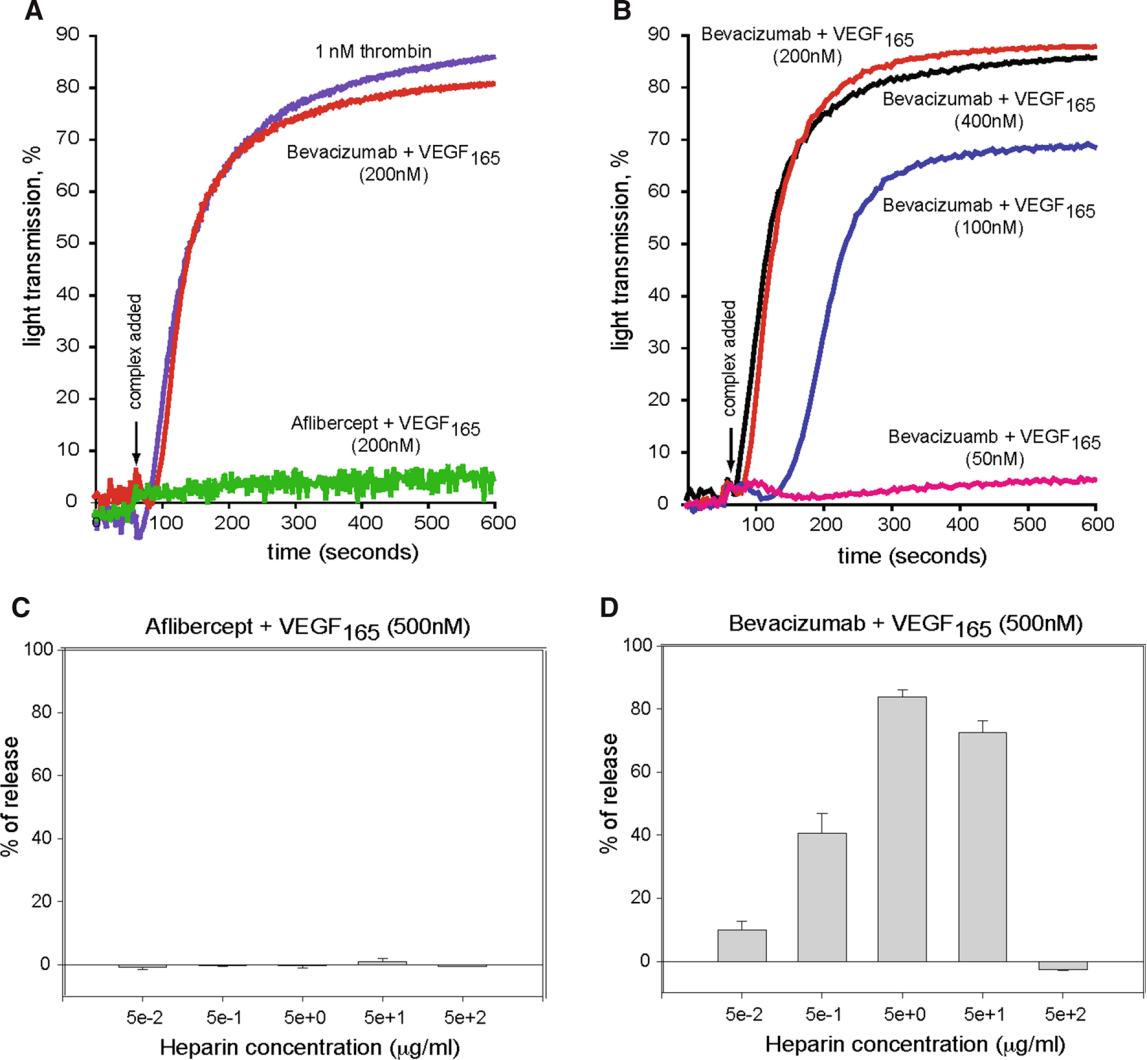
Aflibercept:VEGF_165_ complexes do not activate platelets in vitro. **a** Preformed equal molar (200 nM) aflibercept:VEGF_165_ or bevacizumab:VEGF_165_ complexes were added to primed (1 µM epinephrine), washed platelets containing 200 nM *UFH* unfractionated heparin and percent light transmittance monitored at 600 nm. Thrombin (1 nM, Chrono-PAR) acted as the positive control. **b** A range (400–50 nM) of preformed equal molar bevacizumab:VEGF_165_ complexes were added to primed, washed platelets containing *UFH* unfractionated heparin and percent light transmittance monitored. A similar experiment using aflibercept:VEGF_165_ complexes did not activate platelets (data not shown), and thus, only data for the 200 nM complex (**a**) are shown. Serotonin release was measured from platelets stimulated in the presence of a range of concentrations (0.1, 0.2, 0.5 or 1.0 µM) of UFH with aflibercept:VEGF_165_ complex (**c**) or bevacizumab:VEGF_165_ complex (**d**). Inhibitor:ligand complex concentration was 500 nM

Previous studies have demonstrated that, in the presence of heparin, bevacizumab:VEGF_165_ complexes can activate human platelets via FcγRIIa [22]. The potential for aflibercept-VEGF_165_ complexes to induce FcγRIIa-dependent platelet activation was tested using light aggregometry. Preformed complexes of aflibercept and VEGF_165_ at equal molar ratios in the presence of heparin failed to induce aggregation of human platelets (Fig. 2a). In contrast, preformed complexes of bevacizumab and VEGF_165_ at equal molar ratios in the presence of heparin caused marked aggregation of platelets over a concentration range of 100–400 nM (Fig. 2b). However, increasing the molar excess of bevacizumab (≥fourfold relative to VEGF_165_) results in a loss of platelet activation (data not shown) Bevacizumab:VEGF complexes below 100 nM did not activate platelets. Platelet activation was not detected at concentrations below 100 nM of bevacizumab:VEGF_165_ complex.

Platelet activation was also tested using a serotonin release assay. The combination of aflibercept and VEGF_165_ at equal molar ratios (500 nM each) in the presence of heparin was unable to stimulate serotonin release from platelets (Fig. 2c). However, the presence of bevacizumab and VEGF_165_ at an equal molar ratio of 500 nM each in the presence of heparin induced up to 80 % release of serotonin from platelets (Fig. 2d), consistent with findings from the light aggregometry assays.

### Aflibercept:VEGF_165_ complexes do not induce thrombocytopenia or thrombosis in FcγRIIa transgenic mice

**Fig. 3:**
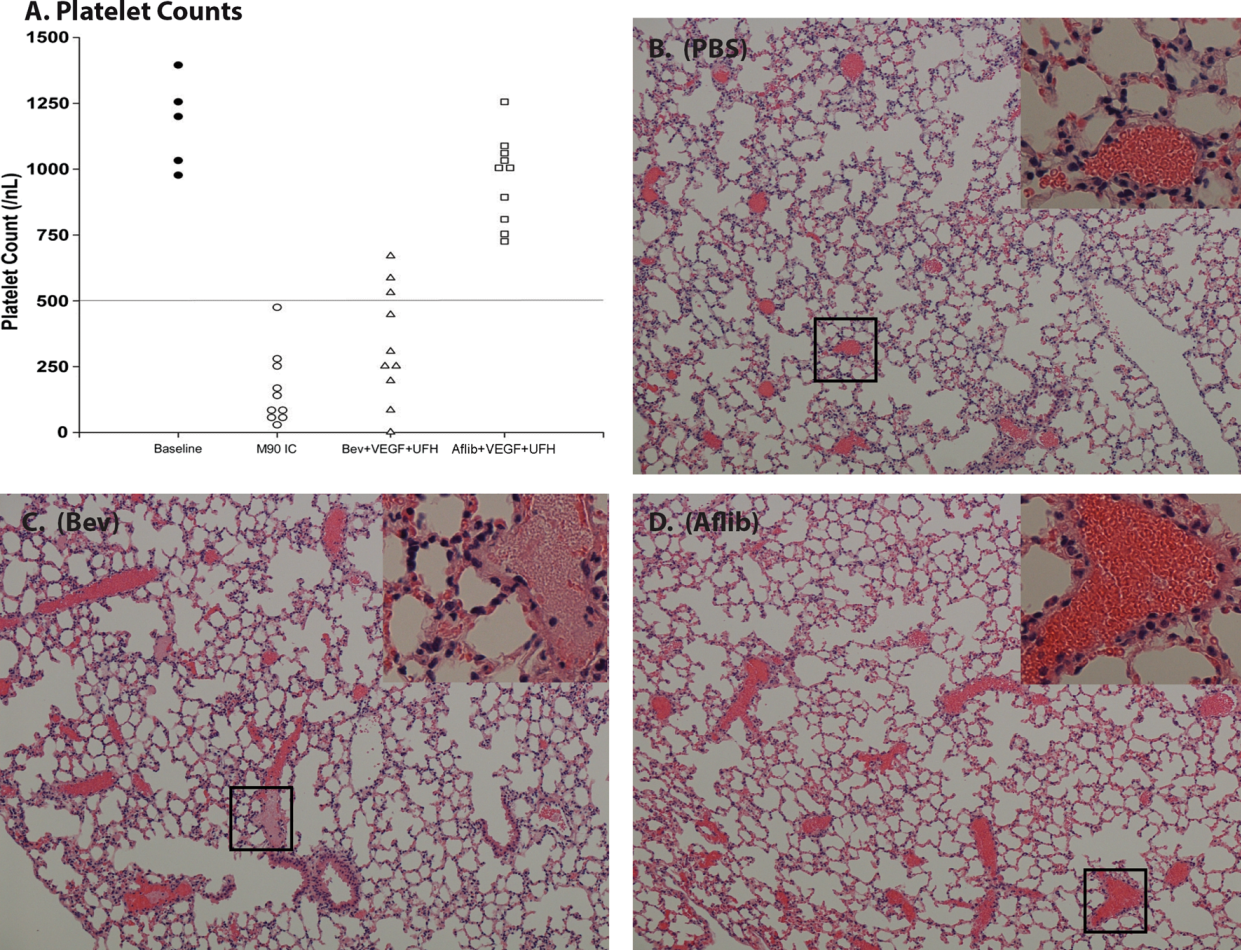
Aflibercept + VEGF_165_ + unfractionated heparin (UFC) complexes do not activate platelets in vivo. B6;SJL-Tg (FcγRIIa)11Mkz (FCGR2A) mice were injected with PBS (*n* = 5) or preformed immune complexes (*n* = 10 per group) via the tail vein. Ten minutes after reagent injection, mice were anesthetized, blood was collected by cardiac puncture, and platelet counts were measured using an automated cell counter. Following blood draws, animals were killed and lungs were dissected, rinsed, and embedded in paraffin. Paraffin blocks were sliced, and cut sections (2 µm thick) were deparaffinized, rehydrated, and stained. Shown are platelet counts per individual FcγRIIa animal by treatment group (**a**) along with representative H&E microscopy sections for PBS (**b**) bevacizumab (Bev) + VEGF_165_ + heparin (**c**) and aflibercept + VEGF_165_ + heparin (**d**). The *horizontal line* in (**a**) represents approximately 60 % of reduction from baseline mean platelet count. Microscopy images were captured at ×200 magnification. *Insets* represent ×700 magnification

Injection of preformed (1:1 molar) bevacizumab:VEGF_165_ complexes along with unfractionated heparin into transgenic mice expressing human FcγRIIa has been reported to cause severe thrombocytopenia and occlusive thrombosis in alveolar capillaries [22]. We sought to determine whether preformed aflibercept:VEGF_165_ complexes in the presence of unfractionated heparin could trigger a similar set of sequelae in human FcγRIIa transgenic mice. Animals receiving aflibercept:VEGF_165_ complexes (1:1 molar ratio) did not exhibit these symptoms (*n* = 10). Platelet counts 10 min following immune complex injection demonstrated thrombocytopenia in animals receiving bevacizumab:VEGF_165_ but not aflibercept:VEGF_165_ complexes (Fig. 3a). Specifically, mice injected with phosphate-buffered saline (PBS; *n* = 5) had baseline platelet counts of (1173 ± 179; mean ± SD), whereas animals receiving bevacizumab + VEGF_165_ had mean platelet counts of 331 ± 217 (with heparin; *P* < 0.001) and 725 ± 303 (without heparin; *P* < 0.003). Mice receiving aflibercept + VEGF_165_ had mean platelet counts of 966 ± 168 (with heparin) and 878 ± 250 (without heparin), which were not statistically different from baseline (PBS) animal counts. Mouse antihuman CD-40 ligand immune complexes [22] served as a positive control and induced severe thrombocytopenia in FcγRIIa transgenic mice with mean platelet counts of 163 ± 137 (*P* < 0.001).

Consistent with the thrombocytopenia, mice injected with bevacizumab:VEGF_165_ complexes developed abundant occlusive thrombi in the pulmonary vasculature (Fig. 3c). In contrast, mice injected with aflibercept:VEGF_165_ complexes failed to develop microvascular thrombosis (Fig. 3d).

### Aflibercept exhibits no significant cell surface binding to ARPE-19 cells or HUVEC

**Fig. 4:**
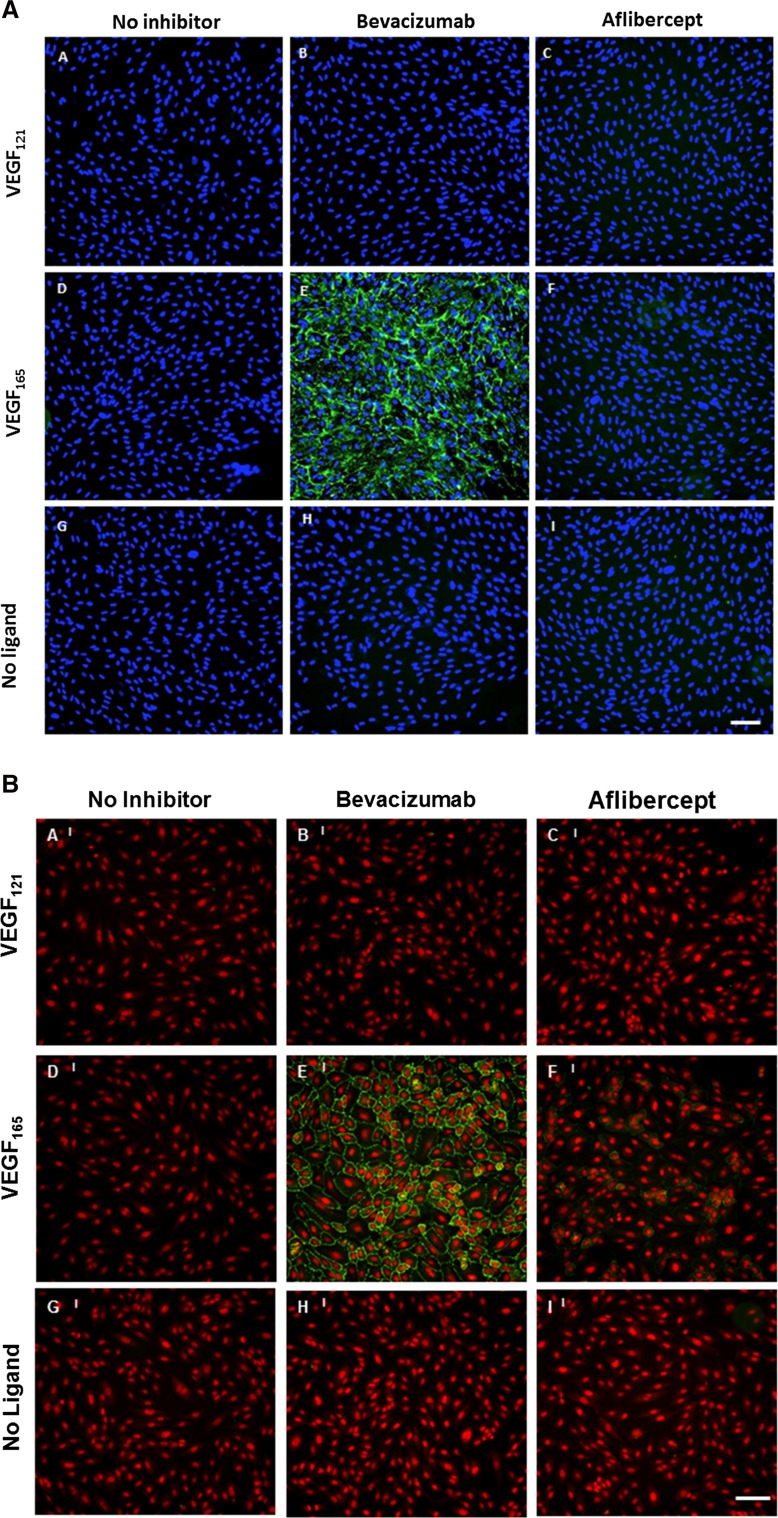
Aflibercept does not exhibit significant cell surface binding to ARPE-19 and HUVEC. Cell surface binding of aflibercept and bevacizumab was evaluated using ARPE-19 (**a**) and HUVEC (**b**). Cells were pre-seeded on collagen-coated 96-well plates. ARPE-19 cells were incubated with 5 nM bevacizumab or aflibercept alone or in the presence of 10 nM VEGF_165_ or 10 nM VEGF_121_ at 37 °C for 1 h. HUVEC were incubated with 15 nM bevacizumab or aflibercept alone or in the presence of 10 nM VEGF_165_ or 10 nM VEGF_121_ at 37 °C for 1 h. Surface-bound inhibitor was detected by incubation with A488-anti-hIgG (Fc-specific) at 4 °C. Cells were washed, fixed with 4 % paraformaldehyde, and counterstained with a nucleic acid counterstain (DAPI for ARPE-19 or DRAQ5 for HUVEC, *red* fluorescence) prior to analysis. Cell surface binding was evaluated with secondary antibody alone (*left column*), bevacizumab (*middle column*) or aflibercept (*right column*) in the presence of VEGF_121_ (**a**–**c**), VEGF_165_ (**d**–**f**) or no ligand (**g**–**i**). *Scale bar* = 50 μm in (**a**) and 100 μm in (**b**)

Cell surface binding of aflibercept and bevacizumab alone or in complex with VEGF was tested using ARPE-19 (Fig. 4a) and HUVEC (Fig. 4b). Binding studies were conducted for both inhibitors with no ligand present or following pre-incubation with VEGF_121_ or VEGF_165_. Binding to ARPE-19 cells was not observed for bevacizumab or aflibercept in complex with VEGF_121_ or in the absence of ligand (Fig. 4a, row 1, 3), but significant ARPE-19 cell surface binding was observed for bevacizumab:VEGF_165_ complexes. Binding was not observed for the aflibercept:VEGF_165_ complex (Fig. 4a, row 2). Similarly, binding to HUVEC was not observed for aflibercept or bevacizumab in complex with VEGF_121_ or in the absence of ligand (Fig. 4b, row 1, 3). However, significant HUVEC cell surface binding was observed for bevacizumab:VEGF_165_ complexes, and only very low levels of surface binding were observed for aflibercept:VEGF_165_ complexes (Fig. 4B, row 2). Complexes binding to the cell surface of both ARPE-19 and HUVEC were found to be dependent on the molar ratio of the inhibitor:VEGF_165_ complex mixtures (Figure S5 and Figure S6). Complexes binding to the cell surface of ARPE-19 were observed at bevacizumab:VEGF_165_ ratios between 0.06:1 and 4.5:1, with the greatest binding seen at a molar ratio of 0.1:1, and complex binding to HUVEC was observed at bevacizumab:VEGF_165_ ratios between 0.5:1 and 13.5:1, with maximal binding observed at a 1.5:1 molar ratio.

### Cell surface binding of bevacizumab:VEGF_165_ complexes is dependent on heparin in ARPE-19 cells and neuropilin-1 in HUVEC

**Fig. 5:**
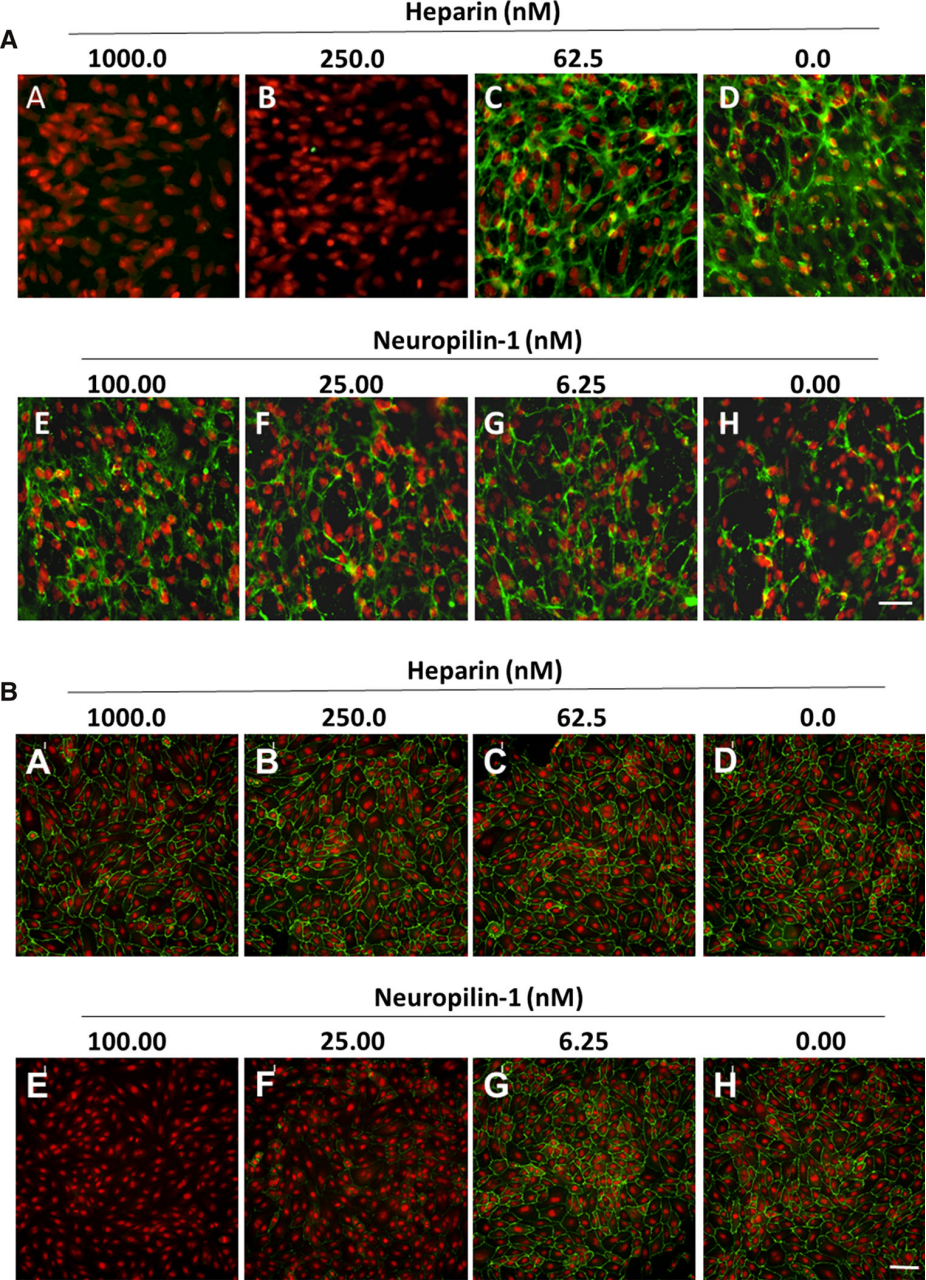
Heparin and neuropilin-1 differentially block bevacizumab cell surface binding. Surface binding to ARPE-19 (**a**) or HUVEC (**b**) cells was evaluated in the presence of soluble heparin and recombinant human neuropilin-1. Cells pre-seeded onto collagen-coated 96-well plates were incubated at 37 °C for 30 min with serial dilutions of soluble heparin or rhNRP-1 pre-complexed with 10 nM VEGF_165_. Bevacizumab was added to the cells to give a final concentration of 15 nM, followed by a 1-h incubation at 37 °C. Surface staining of bevacizumab was detected by incubation with A488 anti-hIgG (*green* fluorescence) at 4 °C. Cells were washed, fixed with 4 % paraformaldehyde, and incubated with a nucleic acid counterstain (DAPI for ARPE-19 or DRAQ5 for HUVEC, *red* fluorescence) prior to analysis. Fluorescence was detected by Molecular Devices ImageXpress High-Content Screening System. *Scale bar* = 50 μm in (**a**) and 100 μm in (**b**)

**Fig. 6:**
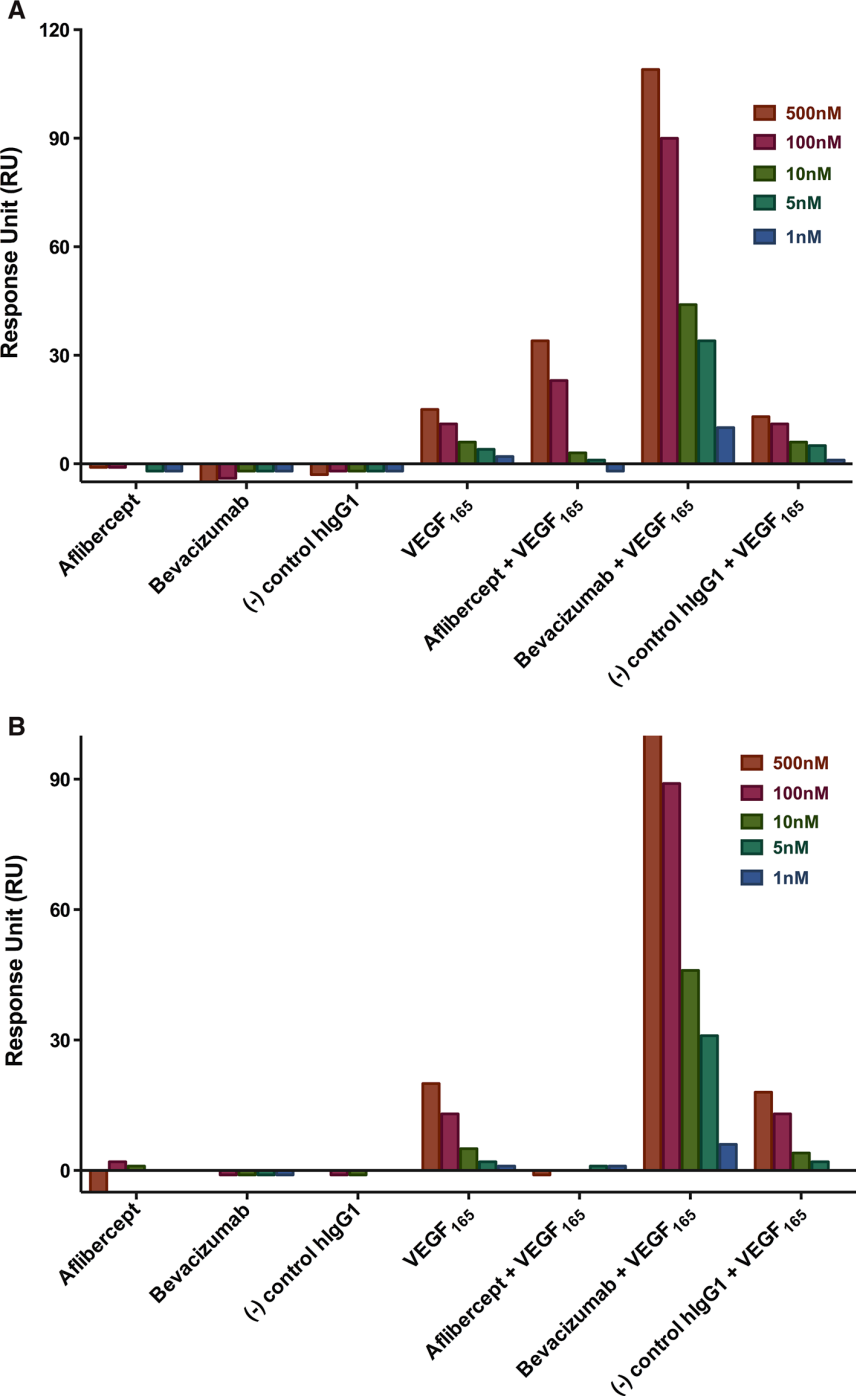
In vitro binding of bevacizumab and aflibercept to NRP1.mFc and heparin–biotin by SPR (Biacore) in the presence of VEGF_165_. **a** Human NRP1.mFc (155 RU) was captured on an anti-mouse Fc-coupled chip surface. The histogram represents bevacizumab and aflibercept at concentrations of 500, 100, 10, 5, and 1 nM either alone or pre-complexed at 1:1 molar ratio with human VEGF_121_ or VEGF_165_. **b** Heparin–biotin (35 RU) was captured on a neutravidin-coupled chip surface. The histogram represents bevacizumab and aflibercept at concentrations of 500, 100, 10, 5, and 1 nM either alone or pre-complexed at 1:1 molar ratio with human VEGF_121_ or VEGF_165_. Representative sensograms of bevacizumab and aflibercept binding to NRP1.mFc and heparin at 5 nM are shown in Supplementary Figure 7

Cell surface binding of bevacizumab:VEGF_165_ complexes was tested in the presence of heparin or recombinant human neuropilin-1 (rhNRP-1). Pre-binding of VEGF_165_ with soluble heparin concentrations of 250 nM or higher prevented binding of bevacizumab:VEGF_165_ complexes to ARPE-19 cells (Fig. 5a). No significant blocking of bevacizumab:VEGF_165_ complex binding to ARPE-19 cells was observed with concentrations of rhNRP-1 of up to 100 nM (Fig. 5a). In contrast, pre-binding of VEGF_165_ with rhNRP-1 (25 nM or higher) prevented binding of bevacizumab:VEGF_165_ complexes to HUVEC (Fig. 5b), while no significant blocking of bevacizumab:VEGF_165_ complex binding to HUVEC was observed in the presence of up to 1000 nM soluble heparin (Fig. 5b). These results are consistent with surface plasmon resonance analysis of bevacizumab:VEGF_165_ and aflibercept:VEGF_165_ in the presence of rhNRP-1 and heparin (Fig. 6a, b, S7A and B). Bevacizumab:VEGF_165_ complexes were found to bind to both rhNRP-1- and heparin-coated surfaces at concentrations as low as 1 nM. Binding of aflibercept:VEGF_165_ complexes to rhNRP-1- and heparin-coated surfaces was only detected at complex concentrations of 100 nM or greater.

### Cell surface binding is dependent on the concentration of endogenous VEGF_165_

**Fig. 7:**
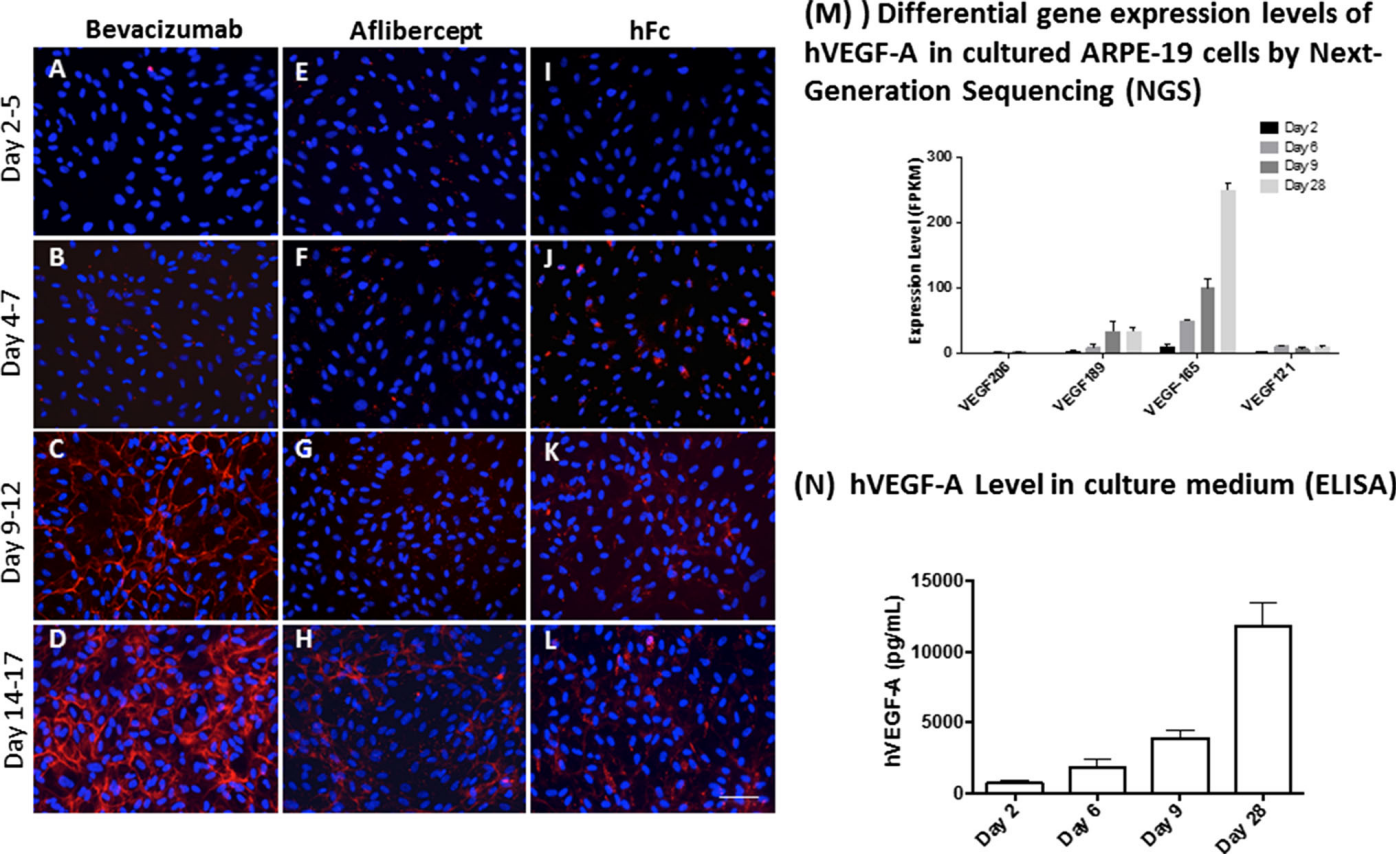
Cell surface binding of bevacizumab is directly proportional to endogenous VEGF_165_ concentration. ARPE-19 cells, cultured on human fibronectin-coated coverslips in six-well plates, were treated with equimolar concentration (1.68 μM) of bevacizumab, aflibercept, or hFc for 3 days starting from different time points (Day 2, 4, 9, and 14) followed by immunofluorescence staining of cell surface-bound inhibitor (*red* fluorescence, detected with mouse antihuman IgG Fc-specific, and secondary Ab, goat anti-mouse IgG-Alexa flour 594. Nuclei were counterstained with DAPI in blue). Cells and culture media at various culture times (Days 2, 6, 9, and 28) were collected for next-generation sequencing and ELISA of VEGF expression levels. Cells treated with bevacizumab (**a**–**d**) showed an increased cell surface binding in confluent ARPE-19 cell culture, coincident with the upregulation of VEGF expression (**m**, **n**). Cells treated with aflibercept (**e**–**h**) or control protein hFc (**i**–**l**) showed minimal binding at all time points. *Scale Bar* = 50 μm

ARPE-19 cells were used to test cell surface binding of bevacizumab and aflibercept in the presence of VEGF endogenously produced by these cells. ARPE-19 cells have been shown to express VEGF after several days in culture [31]. Cells in culture were incubated with aflibercept, bevacizumab, or hFc, for 3 days starting at day 2, 4, 9, and 14, and surface binding was then examined 3 days later. Aflibercept and the hFc control showed minimal surface binding at all time points. In contrast, bevacizumab showed significant cell surface binding at the Day 9–12 and Day 14–17 time points (Fig. 7c–l and 7d–1).

Next-generation sequencing and ELISA binding were used to determine the expression level and identity of the VEGF isoforms present in the culture medium at various time points. VEGF_165_ was found to be the predominant VEGF species present (Fig. 7m, n), with the concentration of this isoform increasing as a function of ARPE-19 time in culture. The highest concentrations of VEGF_165_ were found on the Day 9 and Day 28 time points, consistent with the high level of bevacizumab cell surface binding at later time points. These results closely resemble cell surface binding studies conducted with preformed bevacizumab:VEGF_165_ complexes and suggest that bevacizumab can spontaneously form multimeric complexes in the presence of endogenous VEGF_165_.

## Discussion

Several reports have indicated that bevacizumab, in contrast to ranibizumab, can bind cell surface-bound VEGF on RPE and endothelial cells [27, 32]. Additional studies have provided evidence that bevacizumab not only binds cell-surface-bound VEGF on RPE cells, but that this binding may trigger the complement cascade resulting in cell death [3, 33]. Components of the complement pathway are known to play a role in AMD pathogenesis, although the mechanism has not been clearly defined. Activation of the complement cascade can result in damage to retinal cells as well as surrounding tissue [34], and conversely, complement components can contribute to neuronal homeostasis in the retina [35]. Aflibercept, like bevacizumab, contains an Fc domain; however, several reports have shown that this anti-VEGF agent does not induce changes in cell morphology, induce apoptosis, or decrease cell viability of retinal cells in culture [36, 37].

To further understand what could be driving these observed differences in vitro, we conducted a detailed analysis of bevacizumab and aflibercept when complexed with VEGF by analyzing their stoichiometry of binding, affinity to Fcγ receptors, propensity to activate platelets and ability to bind ARPE-19 and HUVEC. The results of these experiments demonstrate that in contrast to bevacizumab, which can form large multimeric complexes with VEGF, aflibercept forms a homogeneous 1:1 molar complex with VEGF and PlGF. The discrete monomeric complexes of aflibercept:VEGF do not allow avidity-driven Fc to Fcγ receptor interactions as seen with the large heterogeneous complexes that bevacizumab forms with VEGF when both are present at near equal molar ratios. Furthermore, since bevacizumab, unlike aflibercept, does not block heparin binding to VEGF_165_, these multimeric bevacizumab:VEGF complexes, in the presence of heparin, can activate FcγRIIa receptors triggering platelet aggregation. Lastly, this fundamental difference in binding geometry explains the observations that no significant cell binding was observed for aflibercept:VEGF complexes, while the heterogeneous bevacizumab:VEGF complexes bound to the cell surfaces of both ARPE-19 and HUVEC.

This report demonstrates at molar ratios of 5:1 to 1:5, aflibercept to VEGF_165_, aflibercept was observed to form a 1:1 molar complex. In contrast, bevacizumab at the same molar ratios formed high molecular weight complexes with VEGF_165_, in agreement with earlier published work [21]. When the affinity of these observed high molecular weight complexes to high- and low-affinity Fcγ receptors was investigated, we demonstrated that bevacizumab, when bound to either VEGF_121_ or VEGF_165_ at equal molar ratios (~1 µM), had drastically increased *t*_1/2_ values, ranging from ~threefold for the FcγRI receptor to ~177-fold increase for the low-affinity FcγRIIb receptor, compared to bevacizumab alone. In contrast, aflibercept in complex with VEGF_121_ or VEGF_165_ at equal molar ratios (~1 µM) showed no appreciable difference in *t*_1/2_ values relative to aflibercept alone.

Through platelet activation studies, we showed that when heparin was added to bevacizumab:VEGF complexes (100–400 nM), marked aggregation of platelets was observed. Heparin, in this context, allows for optimal FcγRIIa receptor clustering by bringing the bevacizumab:VEGF complexes to the platelet surface [22]. However, when we increased the molar excess of bevacizumab (≥fourfold relative to VEGF_165_), the ability to trigger platelet activation was lost, most likely due to the ability of excess bevacizumab to decrease the size of the multimeric complexes, reducing the avidity between bevacizumab’s Fc domain and the FcγRIIa receptor. In contrast, preformed complexes of aflibercept and VEGF_165_ at equal molar ratios in the presence of heparin failed to induce aggregation of human platelets. Consistent with these results, serotonin release from platelets was not observed for aflibercept:VEGF_165_ complexes but was observed for bevacizumab:VEGF_165_ complexes. These bevacizumab:VEGF_165_ complexes could form when systemic or intravitreal levels of bevacizumab are low, approaching the concentrations of endogenous VEGF.

To study the potential sequelae of bevacizumab’s immune complex formation with VEGF, we incubated bevacizumab:VEGF_165_ with either ARPE-19 cells or HUVEC. At equimolar ratios of bevacizumab to VEGF_165_, the complexes bound to the ARPE-19 cell surface. Indeed, there was significant staining of the preformed bevacizumab:VEGF_165_ complexes at molar ratios from 4.5:1 to 0.06:1. These findings support the earlier work of Klettner et al. [38] which showed that bevacizumab was taken up by primary porcine RPE cells as assessed by confocal laser scanning microscopy and flow cytometry. There was no staining on the cell surface with bevacizumab complexed with VEGF_121_, indicating that the heparin-binding domain and/or the NRP1-binding site was required for immune complex deposition. Very little cell surface staining was observed on either ARPE-19 cell or HUVEC by aflibercept:VEGF_165_ complex over a wide range of molar ratios.

The binding of bevacizumab on the cell surface only when in complex with VEGF_165_ but not VEGF_121_ points to the involvement of the positively charged heparin-binding domain found on VEGF_165_. The heparin-binding domain confers diverse functions on VEGF_165_ [39], including interaction with heparin and neuropilin present on the cell surface. Binding of the bevacizumab:VEGF_165_ complex to ARPE-19 cells was blocked by the addition of heparin but not neuropilin. Conversely, binding of the bevacizumab:VEGF_165_ complex to HUVEC was blocked only by the addition of neuropilin, illustrating that both heparin or neuropilin can anchor these complexes to the cell surface.

To determine whether these immune complexes could form with endogenously produced VEGF, we added either bevacizumab or aflibercept to ARPE-19 cells after allowing VEGF production to proceed over various time periods (2, 4, 9, and 14 days). After quantifying VEGF levels using both ELISA and next-generation sequencing, it was clear that bevacizumab cell surface staining correlated with the levels of VEGF produced. As in the studies using exogenous VEGF, the addition of aflibercept to ARPE-19 cells containing different levels of VEGF did not result in detectable cell surface staining.

In conclusion, we have demonstrated that bevacizumab and aflibercept can exhibit strikingly different binding stoichiometries with VEGF. When the molar concentration of bevacizumab is within approximately tenfold that of VEGF, two molecules of bevacizumab can bind to each VEGF dimer via one of its two Fab arms, leaving the second Fab arm on each bevacizumab free to bind another VEGF dimer. This binding stoichiometry promotes the formation of large, multimeric complexes of bevacizumab which exhibit significantly enhanced binding affinity toward low-affinity Fcγ receptors. Moreover, the heparin- and neuropilin-binding sites of VEGF_165_ are not occluded when bound to bevacizumab, such that heparin/neuropilin-mediated cell surface binding is also enhanced. That large multimeric complexes of bevacizumab:VEGF_165_ exhibit enhanced binding to both heparin/neuropilin and low-affinity Fc receptors on cell surfaces underlies the ability of these complexes to promote platelet aggregation and activation. In contrast, each aflibercept molecule forms a homogenous 1:1 complex with each VEGF dimer at all molar ratios of aflibercept:VEGF tested. This 1:1 binding stoichiometry does not enhance binding to heparin or neuropilin on cell surfaces. In addition, 1:1 aflibercept:VEGF complexes do not bind low-affinity Fcγ receptors more avidly than unbound aflibercept or monomeric IgG1, or cause platelet aggregation and activation. While there is no evidence to link the reported higher gastrointestinal disorders and the mechanism by which bevacizumab binds VEGF, we have shown that differences in binding at high molar ratios can be directly correlated to differences in Fcγ receptor binding, cell surface binding, and platelet activation.

## Materials and methods

### Multi-angle laser light scattering detection coupled to size exclusion chromatography (SEC–MALLS)

The SEC–MALLS system comprised an Agilent 1200 Series HPLC system equipped with an ultraviolet (UV) diode array detector coupled to a Wyatt Technology MiniDawn TREOS laser light scattering (LS) detector and an Optilab REX differential refractometer (RI) detector (Santa Barbara, CA). The detectors were connected in series in the following order: UV–LS–RI. LS and RI detectors were calibrated according to the instructions provided by Wyatt Technology.

Complexes comprising 20 µM VEGF inhibitor (aflibercept [Regeneron] or bevacizumab [Roche]) and 100 μM VEGF_165_ (Regeneron, produced in CHO cells) were prepared separately and incubated for 12 h at ambient temperature to form 1:5 molar ratio inhibitor:ligand complexes. Similarly, 5:1 inhibitor:ligand complexes were prepared with 100 µM VEGF inhibitor and 20 µM VEGF_165_. Complexes comprising PlGF-2 (Regeneron) and VEGF inhibitor (aflibercept or bevacizumab) were prepared in the same manner as the inhibitor:VEGF_165_ complexes. Appropriate amounts of unbound inhibitor (20 µM) or ligand (40 µM) were also injected separately into a pre-equilibrated Superose 12 10/300 GL (GE Healthcare) size exclusion column. The mobile phase was 10 mM phosphate, 500 mM NaCl, pH 7.0 with a flow rate of 0.3 mL/min. Molar masses of free ligands, free inhibitor, and inhibitor:ligand complexes were determined using ASTRA software (Wyatt Technology) as previously described [40]. The standard deviation calculated from bovine serum albumin (BSA) standard samples was within 2 % in different SEC–MALLS experiments.

### Surface plasmon resonance kinetic binding assay

SPR experiments were performed on a Biacore 3000 instrument using a dextran-coated (CM4 or CM5) chip at 25 °C. The running buffer was filtered HBS-T (10 mM Hepes, 150 mM NaCl, 3.4 mM EDTA, 0.05 % polysorbate 20, pH 7.4). A capture sensor surface was prepared by covalently immobilizing anti-histidine monoclonal antibody (His capture kit, GE Healthcare), anti-mouse Fc (Mouse antibody capture kit, GE Healthcare), or neutravidin (Thermo Fisher Scientific Inc.) to the chip surface using 1-Ethyl-3-[3-dimethylaminopropyl]carbodiimide hydrochloride/*N*-hydroxysuccinimide (EDC/NHS) coupling chemistry. Following surface activation, capturing reagents in coupling buffer (0.1 M acetate buffer, pH 4.5) were injected over the activated chip surface until the binding signals in resonance unit (RU) reached about 3500 RU for the anti-histidine monoclonal antibody surface, 1500 RU for the anti-mouse Fc polyclonal antibody, and 5000 RU for the neutravidin, respectively. The surfaces were then washed and treated with 10 mM glycine–HCl at pH 1.5 to remove uncoupled residual proteins. The various his-tagged or myc–myc His-tagged Fcγ receptor family members, mFc-tagged human NRP1 (Regeneron), and biotinylated heparin (Sigma-Aldrich) were injected over the corresponding capture surfaces to a low density at approximate 200 RU in the running buffer with a concentration range between 5 and 10 µg/mL. The His-tagged FcγRIIA_R131_, FcγRIIB/C, FcγRIIIB, and FcγRI were purchased from R&D Systems, and myc–myc His-tagged FcγRIIIA_F176_, and FcγRIIIA_V176_ were generated at Regeneron.

Following the capture step, bevacizumab (Roche) or aflibercept (Regeneron) solutions with and without equimolar concentrations of VEGF_165_ (Regeneron) or VEGF_121_ (R&D Systems, produced in *E. coli*) (from 1.0 µM to 31.25 nM in a twofold serial dilutions) were individually injected over the various Fcγ receptor, human NRP1.mFc, and biotin–heparin surfaces, and the real-time binding signals were recorded. The specific binding sensorgrams were obtained using a double-referencing procedure as described by Myszka et al. [41] by subtracting the binding signal on the blank capture surface and sample buffer run. The binding data were then processed and analyzed using Scrubber software (version 2.0, BioLogic Software), and the dissociation rate constant (*k*_d_) and dissociation half-life *t*_1/2_ were calculated.

### Platelet aggregation

#### Washed platelet preparation

Platelet concentrate was obtained from the NY Blood Center and used before expiration. Washed platelet suspensions were prepared from pooled platelets obtained from citrated phosphate dextrose blood using the method of Cazenave et al. [42]. Approximately 20 mL of platelet concentrate was centrifuged (2200*g*, 25 °C) for 12 min. The supernatant was removed, and 20 mL of modified Tyrode’s buffer (137 mM NaCl, 2.7 mM KCl, 12 mM NaHCO_3_, 0.42 mM NaH_2_PO_4_, 2 mM MgCl_2_, 5 mM HEPES, 5.55 mM dextrose, pH 6.2) containing 1.5 U/mL apyrase Grade VII (Sigma-Aldrich) and 0.5 µM prostacyclin *I*_2_ (Cayman Chemical) was added. The platelets were resuspended and allowed to rest for 10 min at 37 °C. Following this, another 0.5 µM of prostacyclin *I*_2_ was added, and the platelets were centrifuged (1900*g*, 25 °C) for 8 min. The supernatant was removed, and another 20 mL of modified Tyrode’s buffer containing 1.5 U/mL apyrase Grade VII and 0.5 µM prostacyclin *I*_2_ was used to resuspend the platelets. The platelets were allowed to rest for 10 min at 37 °C. After this second wash step, 0.5 µM of prostacyclin was added and the platelets were centrifuged (1900*g*, 25 °C) for 8 min. The supernatant was removed, and 40 mL of Tyrode’s buffer containing calcium (Tyrode’s buffer with 2 mM CaCl_2_, pH 7.35) was added. The platelets were resuspended and diluted to 300,000 cells/µL using the same buffer. Using this procedure, the washed platelet suspension was stable for 4 h when stored at 37 °C.

#### Platelet aggregometry

Platelet aggregation was monitored at an absorbance of 600 nm using a Beckman DU7400 spectrophotometer fitted with a homemade thermostatic stirring chamber. In typical experiments, 1.8 mL of platelets was added to 3-mL plastic cuvettes containing a stir bar designed for spectrophotometer cells (Fisher Scientific). The platelets, under constant stirring, were allowed to reach thermo-equilibrium for 5 min. Then, 100 µL of PBS containing agonist (epinephrine, Sigma-Aldrich) and unfractionated heparin (Sigma-Aldrich) was added. After 1 min, data collection was begun and an additional 100 µL of PBS containing the bevacizumab:VEGF or aflibercept:VEGF complex was added. Platelet aggregation was typically monitored for 10–15 min. Plots of percent aggregation were calculated by taking the initial average absorbance of the platelets (~1.3–1.4 AU) as 0 % aggregation and an absorbance of zero as 100 % aggregation.

### Serotonin release assay

In vitro platelet activation was studied using a serotonin release model described by Meyer et al. [22]. Human platelets were loaded with ^14^C-serotonin (PerkinElmer), then washed, and incubated at 22 °C with the test complex for 1 h under constant stirring. Released serotonin was counted in the supernatant and expressed as a percentage of the total serotonin loaded into the platelets. To create bevacizumab:VEGF_165_ or aflibercept:VEGF_165_ complexes, VEGF_165_ (R&D Systems) and bevacizumab (Roche) or aflibercept (Regeneron) were coincubated in PBS at equimolar concentrations (2.5 µM) for 15 min at 22 °C. The complex was then added to ^14^C-serotonin-loaded platelets in the presence of heparin (Sigma-Aldrich). Complex was added to platelets to obtain a final concentration of 0.1, 0.2, 0.5, or 1 µM and tested in the presence of a range of unfractionated heparin concentrations (0.5-500 µg/mL).

### Assessment of platelet-activating activity in FcγRIIa transgenic mice

#### Experimental animals

B6;SJL-Tg (FcγRIIa)11Mkz (FCGR2A) mice were obtained from Jackson Laboratories and bred in the Animal Care Facility at Florida Hospital, Orlando, in compliance with approved Institutional Animal Care and Use Committee (IACUC) guidelines and protocols. All experiments were performed in 8- to 15-week-old mice weighing between 16 and 20 g. All animals were genotyped and confirmed positive for the FcγRIIa transgene according to Jackson Laboratories PCR Protocols. Some animals were injected with PBS (vehicle containing no immune complex components) in order to establish baseline platelet counts, as described below.

#### Mouse blood sampling, platelet counting, and intravenous injection procedures

For IV injections, mice were immobilized in a standard restrainer and warmed using a heat lamp for 2 min to dilate the tail vein. Immune complexes were preassembled by mixing antibody + antigen at a 1:1 molar ratio (126 µg M90 [Florida Hospital] + 45 µg CD154 [Peprotech], or 35 µg VEGF [Peprotech, produced in *E. coli*] + either 126 µg bevacizumab [Genentech] or 92 µg aflibercept [Regeneron] ± heparin [Sigma-Aldrich] in PBS). Immune complexes were injected slowly in a 200-µL bolus into the lateral tail vein. Animals were then removed from their restraining device and placed in an open observation cage for monitoring. Ten minutes after reagent injection, mice were anesthetized by isoflurane inhalation. Approximately 500 µL of blood was collected by cardiac puncture via 27-gauge needles into syringes containing 50 µL of acid citrate dextrose (ACD). The blood was immediately placed in a polypropylene collection tube containing 50 µL of ACD, and platelet counts were measured using an automated cell counter. Platelet counts were adjusted for citrate solution volume. All animals were observed for signs of distress throughout the test procedure, specifically for rapid shallow breathing, hunched posture, and signs of decreased or dysfunctional locomotor activity. The combination of these symptoms is often referred to as a “shock” or “thrombotic” phenotype. The observation of such symptoms was recorded along with platelet counts per animal. Together, platelet counts and signs of physical distress constitute the primary data in this animal model.

#### Histochemical analysis of lung sections

Following blood draws, animals were killed by CO_2_ inhalation. Mouse lungs were dissected *en bloc* from isofluorane-anesthetized animals not subjected to cardiac puncture. Lungs were rinsed in PBS and immediately placed in 10 % formalin–PBS for at least 24 h prior to paraffin embedding. Histopathology laboratory technicians cut some distance into the paraffin block (lung tissue) prior to initial sectioning. Cut sections (2 µm thick) were deparaffinized and rehydrated and then processed by an automated H&E staining process at the Florida Hospital main Histopathology Laboratory. Lungs sections from each group were analyzed microscopically for evidence of thrombosis by two independent observers. Also, reference lungs were harvested from baseline animals and processed identically as described above. Fields were captured from each section using a Nikon Eclipse 80i upright microscope equipped with Plan Fluor objectives (10×, *NA* = 0.3, DIC; 20×, *NA* = 0.5, DIC; 40×, *NA* = 0.75, DIC; and 60×, *NA* = 0.85, not DIC), a DC-Fi1 digital camera, and a DS-L2 image capture device. The eyepiece and camera had a collective magnification of 10×, resulting in final image magnifications of 100, 200, 400, or 600×, respectively, with each objective lens. The capture device was activated by pressing a “Capture” button. The image capture device was set by integrated software to automatically adjust for white balance. Observers were instructed to adjust lamp intensity and stage diaphragm (aperture) settings as needed per image; thus, variations in brightness, contrast, and color hue may be observed in some images. Images were converted from TIF format to JPEG and processed in Adobe Photoshop Elements (v8) *without region-specific modifications* (i.e., all changes were global, and no region-specific change was made to any image).

### Statistical analysis

Statistical analysis was performed using SigmaPlot v11 software. The distributions of experimental data sets for mouse platelet counts were determined by normal probability analysis. All groups were found to be normally distributed and of equal variance. Therefore, differences between groups were analyzed by one-way ANOVA using the raw data (rather than the means and standard deviations). The statistical test used was the multiple comparisons versus control group Bonferroni *t* test (alpha = 0.050:1.000).

### Cell surface binding of VEGF inhibitors to ARPE-19 cells in the presence of exogenous VEGF

Confluent ARPE-19 cells (ATCC) on 96-well collagen-coated plates (Greiner CellCoat) were incubated with 5 nM bevacizumab (Roche), or aflibercept (Regeneron) alone or in the presence of 10 nM VEGF_165_ (Regeneron) or 10 nM VEGF_121_ (R&D Systems) at 37 °C for 1 h. Cell surface-bound VEGF inhibitors were detected with Alexa 488 goat antihuman IgG (H + L) Fab fragment (A488-anti-hIgG, Jackson Immunoresearch), followed by fixation with 4 % paraformaldehyde and counterstaining with DAPI. Fluorescence was detected using a Molecular Devices ImageXpress Micro XL High-Content Imaging System equipped with a Nikon 10× Plan Fluor WD objective lens (NA 0.30). All images were acquired with a Molecular Devices 1.4 megapixel cooled CCD camera using MetaXepress^®^ High-Content Image Acquisition and Analysis Software and analyzed using PerkinElmer Columbus Image Data Storage and Analysis System. Figures were made in Adobe Photoshop. No adjustments were made to the original images.

### Cell surface binding of VEGF inhibitors to HUVEC in the presence of exogenous VEGF

HUVEC (Vec Technologies) were seeded into 96-well collagen-coated plates (Greiner CellCoat) at a concentration of 20,000 cells/well in complete medium (Vec Technologies) and incubated overnight at 37 °C. The cells were washed 2× in PBS, followed by sequential addition of 10 nM VEGF_165_ or 10 nM VEGF_121_ and serial dilutions of bevacizumab or aflibercept in assay medium (2 % FBS in PBS). Control wells containing ligand, bevacizumab, or aflibercept alone were also evaluated. After a 2-h incubation at 37 °C, the cells were washed 2× in PBS. The presence of cell-bound inhibitor was determined by incubation with 1 μg/mL A488-anti-hIgG (Jackson) in assay medium for 30 min at 4 °C. The cells were washed 2× in PBS and then fixed in 3.7 % formaldehyde in PBS for 20 min at room temperature followed by a final wash with PBS. Cells were counterstained with Draq5 (Cell Signaling Technologies) following the manufacturer’s instructions and imaged using Molecular Devices ImagExpress Micro XL High-Content Imaging System equipped with a Nikon 20X S Plan Fluor EL WD objective lens (NA 0.45; WD 8.2–6.9 mm). All images were acquired with a Molecular Devices 1.4 megapixel cooled CCD camera using MetaXepress^®^ High-Content Image Acquisition and Analysis Software and analyzed using PerkinElmer Columbus Image Data Storage and Analysis System. Figures were made in Adobe Photoshop. No adjustments were made to the original images.

### Heparin or neuropilin-1 blockade of VEGF_165_ bridging in ARPE-19 cells

ARPE-19 cells (ATCC) were incubated for 30 min with serial dilutions of soluble heparin (Sigma-Aldrich; starting from 1000 nM, 1:2) or rhNRP-1-mFc (Regeneron; starting from 100 nM, 1:2) pre-incubated with 10 nM VEGF_165_ (Regeneron). Bevacizumab was added to the cells (final concentration 10 nM), followed by a 1-h incubation at 37 °C. Surface staining of bevacizumab was detected with A488-anti-hIgG (Jackson Immunoresearch), followed by fixation in 4 % paraformaldehyde and counterstaining with DAPI. Fluorescence was detected by a Molecular Devices ImageXpress Micro XL High-Content Imaging System equipped with a Nikon 20x Plan Fluor ELWD objective lens (NA 0.45; WD 8.2–6.9 mm). All images were acquired with a Molecular Devices 1.4 megapixel cooled CCD camera using MetaXepress^®^ High-Content Image Acquisition and Analysis Software and analyzed using PerkinElmer Columbus Image Data Storage and Analysis System. Figures were made in Adobe Photoshop. No adjustments were made to the original images.

### Heparin or neuropilin-1 blockade of VEGF_165_ bridging in HUVEC

Serial dilutions of heparin (Sigma-Aldrich) or rhNRP-1-mFc (Regeneron) were premixed with 10 nM VEGF_165_. The mixture was then added to HUVEC plated as described above and incubated for 30 min at 37 °C. A constant concentration of 15 nM bevacizumab was then added to cells, followed by a 1-h incubation at 37 °C. The cells were washed, detected with 1 μg/mL A488anti-hIgG (Jackson), counterstained with Draq5 (Cell Signaling Technologies), and imaged using Molecular Devices ImagExpress Micro XL High-Content Imaging System equipped with a Nikon 20x S Plan Fluor EL WD objective lens (NA 0.45; WD 8.2–6.9 mm). All images were acquired with a Molecular Devices 1.4 megapixel cooled CCD camera using MetaXepress^®^ High-Content Image Acquisition and Analysis Software and analyzed using PerkinElmer Columbus Image Data Storage and Analysis System. Figures were made in Adobe Photoshop. No adjustments were made to the original images.

### Cell surface binding of VEGF inhibitors to APRE-19 cells in the presence of endogenous VEGF

ARPE-19 cells (ATCC), cultured on human fibronectin (Corning)-coated coverslips in six-well plates, were treated with equimolar concentration (1.68 μM) of bevacizumab (Roche), aflibercept (Regeneron), or hFc (Regeneron) for 3 days starting from different time points (Days 2, 4, 9, and 14) followed by immunofluorescence staining of cell surface-bound inhibitor [red fluorescence, detected with mouse antihuman IgG Fc-specific, and secondary Ab, goat anti-mouse IgG(h + L)-Alexa Fluor 594 (Life Technologies)]. Nuclei were counterstained with DAPI in blue. Fluorescence was detected in ProLong^®^ Gold antifade reagent (Life Technologies) using a Nikon Eclipse 80i microscope equipped with a Nikon Plan APO 20x objective lens (NA 0.75). All images were acquired with a Diagnostic Instruments Spot Camera using Diagnostic Instruments Spot 4.7 software and analyzed using PerkinElmer Columbus Image Data Storage and Analysis System. Figures were made in Adobe Photoshop. The photomerge function was used to merge blue and red channel images.

Cells and culture media at various culture times (Days 2, 6, 9, and 28) were collected for next-generation sequencing (Illumina Hiseq 2000) and ELISA (R&D Systems) of VEGF expression levels.

## Electronic supplementary material

Below is the link to the electronic supplementary material.

Supplementary material 1 (DOC 3820 kb)
