# Supplementary material for: Aflibercept exhibits VEGF binding stoichiometry distinct from bevacizumab and does not support formation of immune-like complexes
Source: Angiogenesis. 2016 May 27;19:389–406. doi: 10.1007/s10456-016-9515-8 (PMC4930479; doi:10.1007/s10456-016-9515-8)
Supplement: Supplementary file 1 — Supplementary material 1 (DOC 3820 kb) [file 10456_2016_9515_MOESM1_ESM.doc]

**Supplementary Material**

**
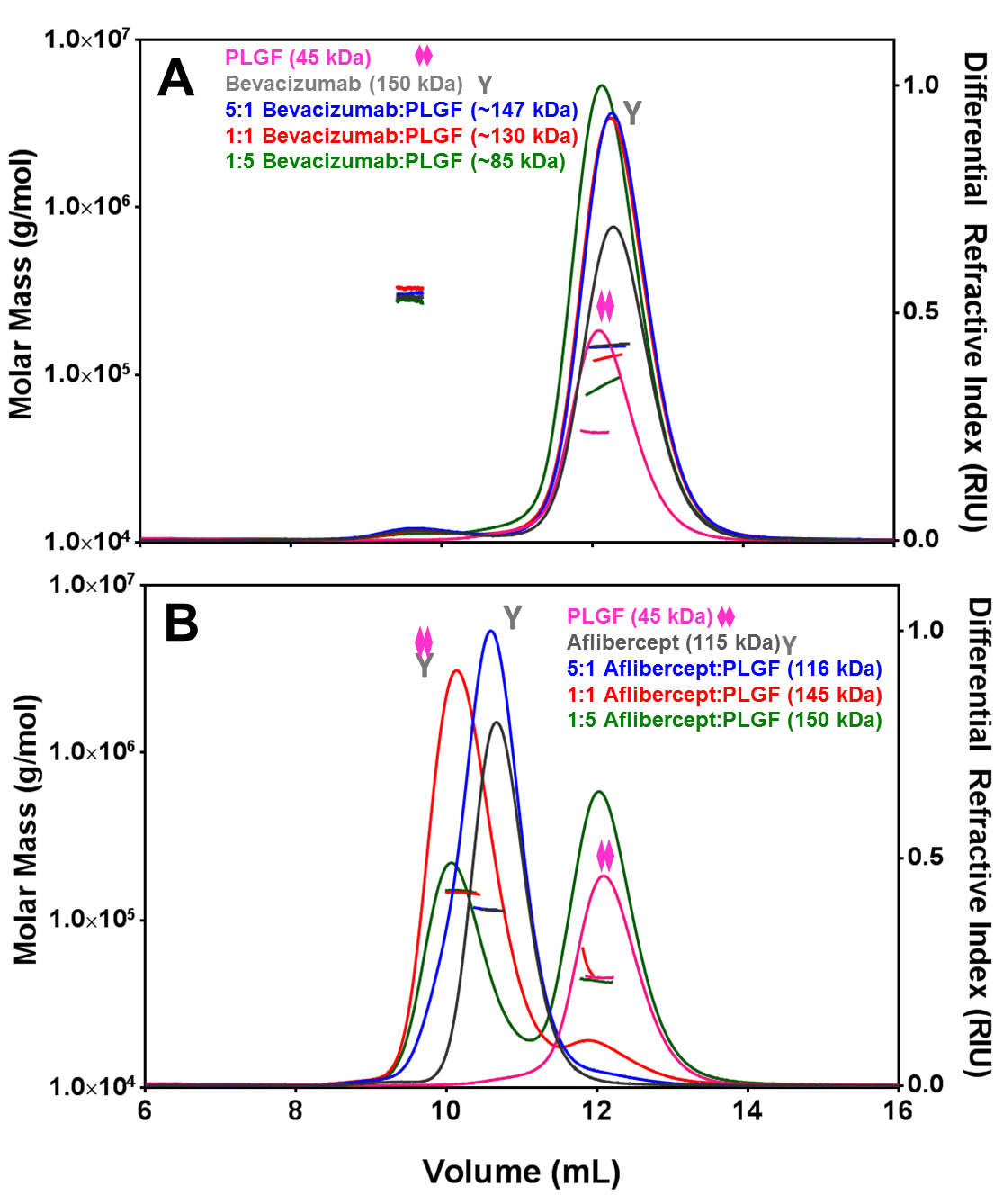
**

**Supplementary Figure 1: Aflibercept forms 1:1 complexes with PlGF-2.** The molar masses of aflibercept:PlGF-2 and bevacizumab:PlGF-2 complexes were analyzed by multi-angle laser light scattering detection coupled to size exclusion chromatography. The differential refractive index as a function of elution volume is shown for each sample and the measured molar masses of peaks are indicated. Cartoons of free PlGF-2 and complexes of aflibercept or bevacizumab bound to PlGF-2 are shown. Mixtures of PlGF-2 with bevacizumab (A) or aflibercept (B) at various molar ratios were incubated for 12 hours at ambient temperature. Following incubation, the samples were kept at 4°C in the autosampler prior to injection (~100-200μg per sample) onto a Superose 12 column pre-equilibrated in 10 mM Phosphate containing 500mM NaCl buffer (pH 7.0) with a flow rate of 0.3mL/min. Chromatograms of PlGF-2 and bevacizumab or aflibercept are superimposed to indicate the elution profiles of the unbound proteins.

**
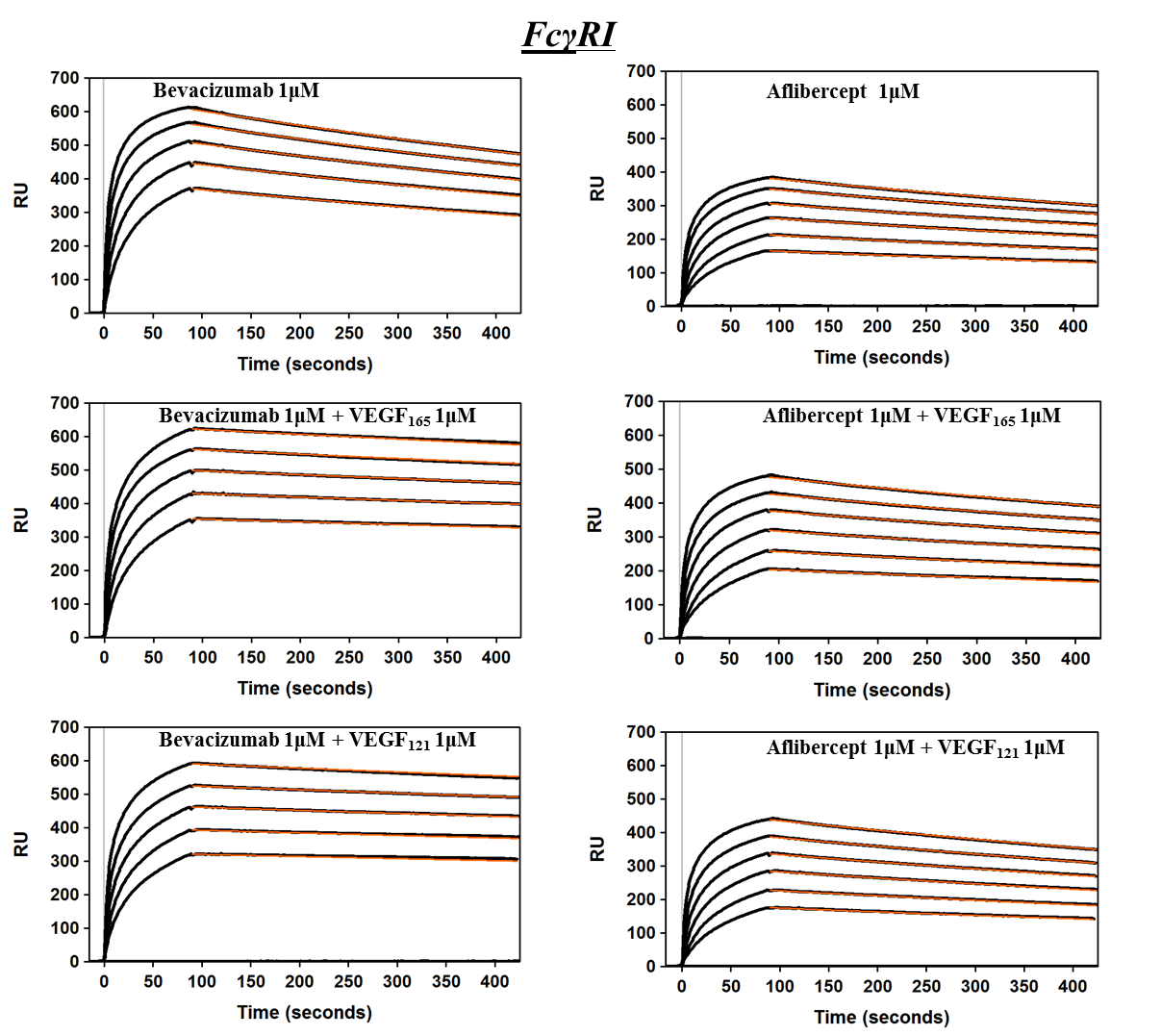
**

**Supplementary Figure 2.** Binding sensorgrams of bevacizumab and aflibercept to human FcγRI in the absence or presence of VEGF-A. The expected high-affinity binding for IgG1-Fc containing molecules such as bevacizumab and aflibercept is observed. The addition of VEGF to either bevacizumab or aflibercept does not significantly alter the observed t_1/2_ values. Bevacizumab:VEGF and aflibercept:VEGF complexes were formed by adding the appropriate VEGF-A isoform at equal molar amounts and allowing the mixture to equilibrate for approximately 2hr at room temperature before SPR-Biacore analysis. Representative FcγRI binding sensorgrams of bevacizumab and aflibercept in the absence or presence of VEGF at multiple concentrations (1, 0.5, 0.25, 0.125, 0.0625 and 0.03125μM) are shown as black lines. The data were only fitted to *k_d_* (determined by monitoring dissociation for up to 5 minutes) using Scrubber 2.0c. Kinetic fits from the analyses are overlaid on the binding data in red.


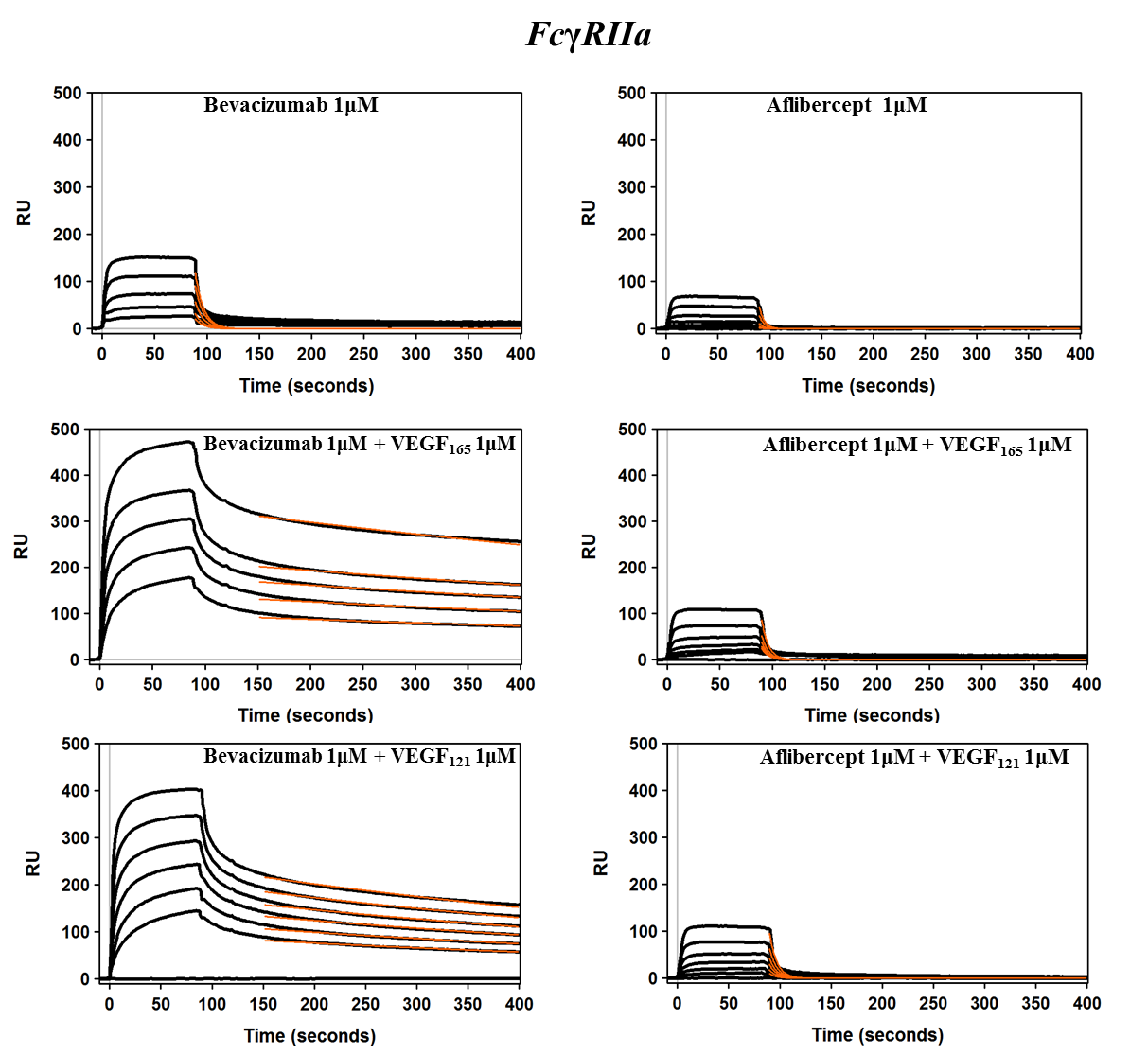

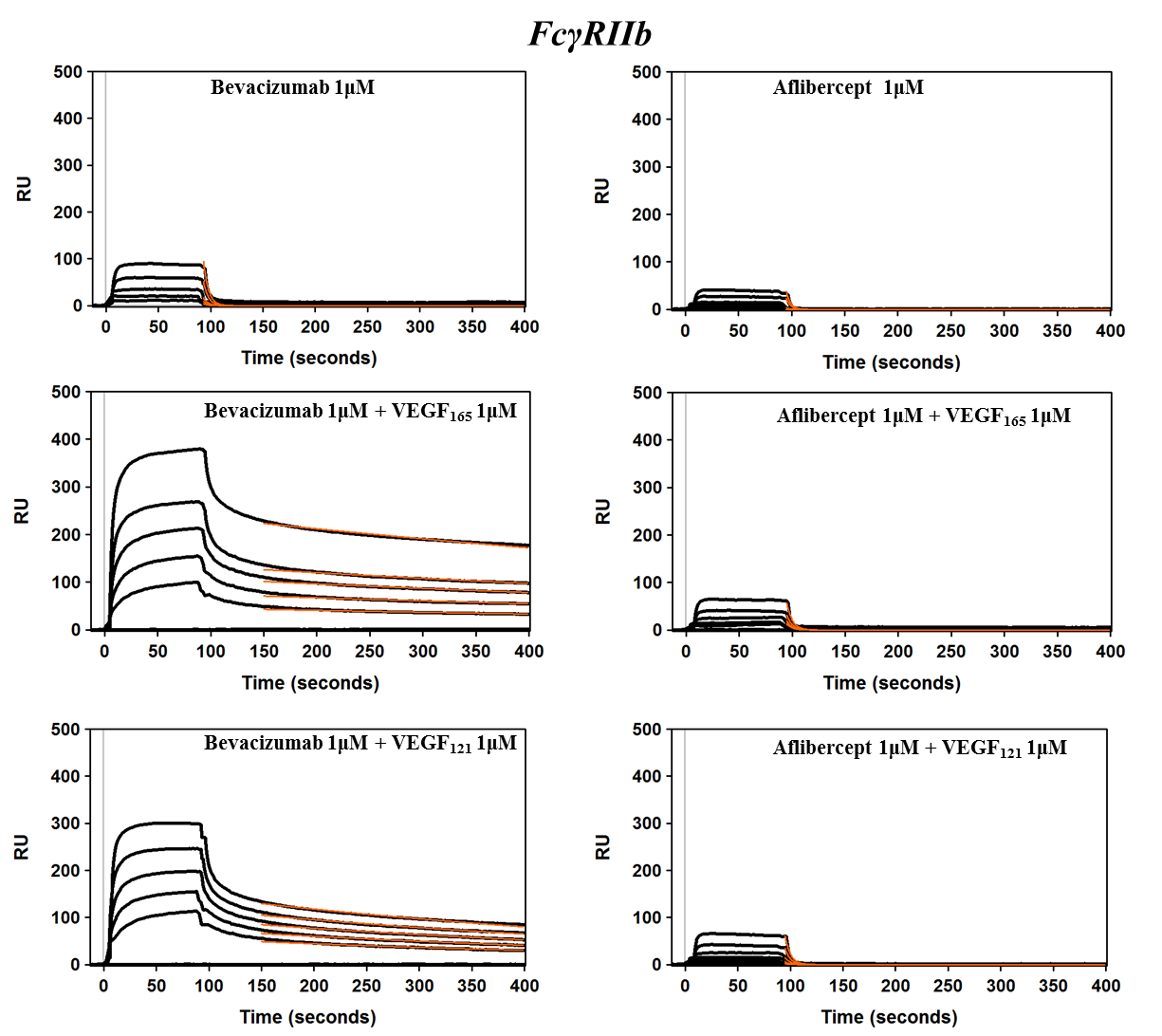


**Supplementary Figure 3.** Binding sensorgrams of bevacizumab and aflibercept to human FcγRIIa and FcγRIIb in the absence or presence of VEGF-A. The expected low affinity (steady-state) binding for IgG1-Fc containing molecules such as bevacizumab and aflibercept is observed. Contrary to aflibercept, the addition of VEGF to bevacizumab significantly prolongs the observed t_1/2_ values. Bevacizumab:VEGF and aflibercept:VEGF complexes were formed by adding equal molar amounts of VEGF-A isoform and allowing the mixture to equilibrate for approximately 2hr at room temperature before SPR-Biacore analysis. The representative FcγRIIa and FcγRIIb binding sensorgrams of bevacizumab and aflibercept in the absence or presence of VEGF at multiple concentrations (1, 0.5, 0.25, 0.125, 0.0625 and 0.03125μM) are shown as black lines. The multimerization of bevacizumab:VEGF complexes created biphasic binding sensogram. The data from 150 to 400 second represent the avidity interaction on the dissociation phase which were fitted to calculate the dissociate rate constant *k_d_* using Scrubber 2.0c. Kinetic fits (in red) from the analyses are overlaid on the binding data.

**Supplementary Figure 4** Binding sensorgrams of bevacizumab and aflibercept to human FcγRIIIa(176V), FcγRIIIa(176F), and FcγRIIIb in the absence or presence of VEGF-A. FcγRIIIa(176V) (221 RU), FcγRIIIa(176F) (217 RU), and FcγRIIIb (244 RU). The expected low affinity (steady-state) binding for IgG1-Fc containing molecules such as bevacizumab and aflibercept is observed. Contrary to aflibercept, the addition of VEGF to bevacizumab significantly prolongs the observed t_1/2_ values. Bevacizumab:VEGF and aflibercept:VEGF complexes were formed by adding the equal molar amounts of VEGF-A isoform at and allowing the mixture to equilibrate for approximately 2 hr at room temperature before SPR-Biacore analysis. The representative FcγRIIIa(176V), FcγRIIIa(176F), and FcγRIIIb binding sensorgrams of bevacizumab and aflibercept in the absence or presence of VEGF at multiple concentrations (1, 0.5, 0.25, 0.125, 0.0625 and 0.03125μM) are shown as black lines. The multimerization of bevacizumab:VEGF complexes created biphasic binding sensogram. The data from 110 to 400 second represents the avidity interaction on the dissociation phase which were fitted to calculate the dissociate rate constant *k_d_* using Scrubber 2.0c, and the kinetic fits (in red) from the analyses are overlaid on the binding data (Myszka, 1999).


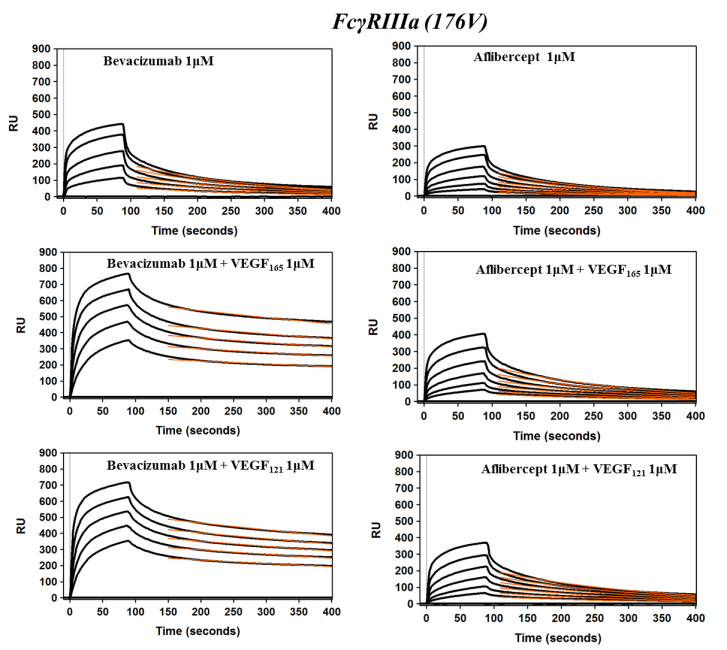

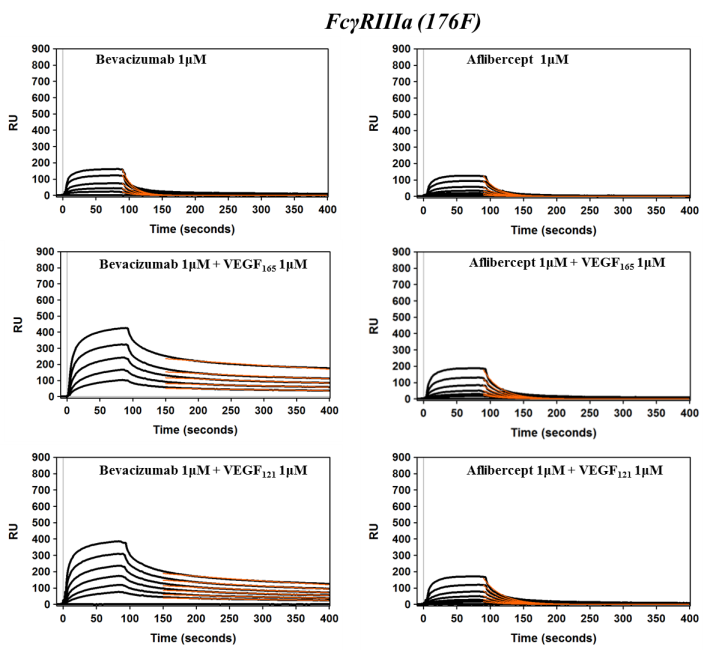

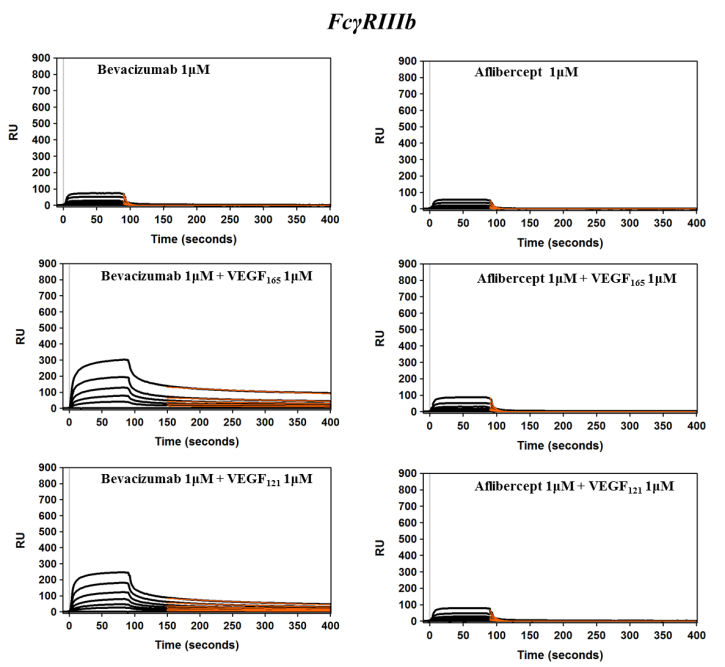


**
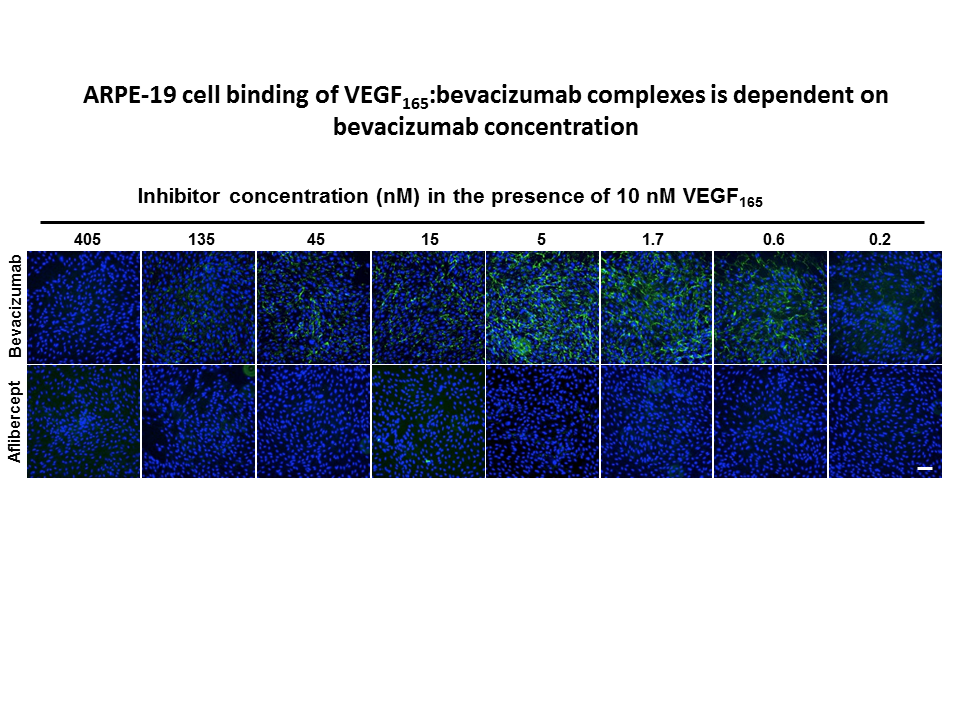
**

**Supplementary Figure 5: Binding of bevacizumab to ARPE-19 cells is concentration dependent.** ARPE-19 cells were incubated with serial dilutions (starting from 405nM, 1:3 dilutions) of bevacizumab (top row) or aflibercept (bottom row) in the presence of 10nM VEGF_165_ at 37°C. Surface bound inhibitor complexed with VEGF_165_ was detected by incubation with A488-anti-hIgG (green fluorescence) at 4°C. Cells were washed, fixed with 4% paraformaldehyde and incubated with a nucleic acid counterstain (DAPI, blue fluorescence) prior to analysis. Significant cell surface binding on ARPE-19 cells was present with bevacizumab from 45nM to 0.6nM + 10nM VEGF_165_. No binding was observed at same relative concentrations with aflibercept + VEGF_165_. Scale bar = 50μm.

**
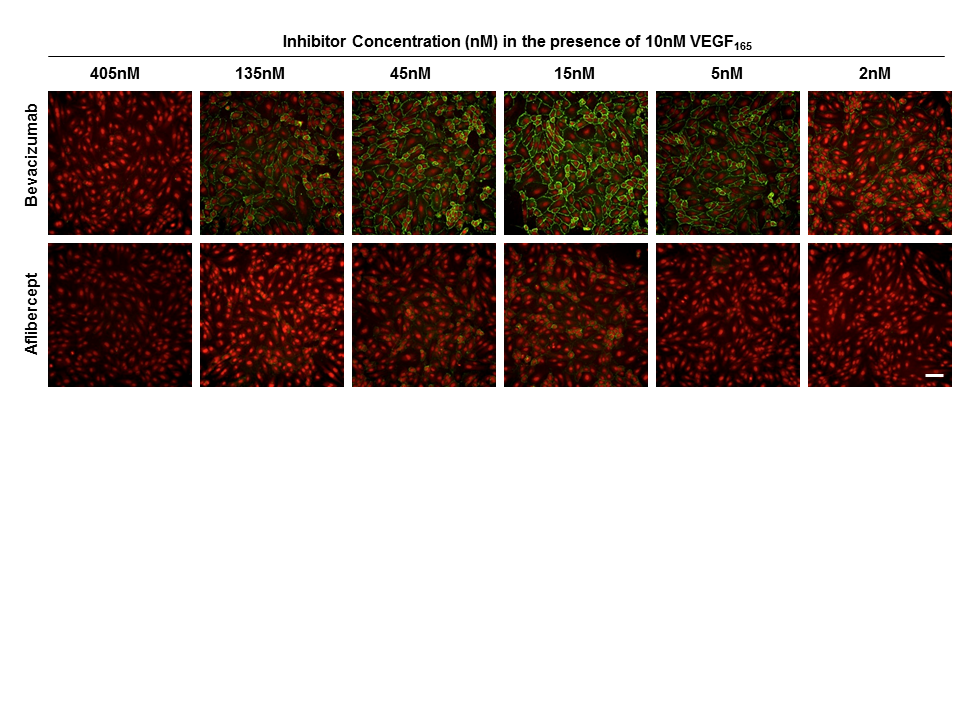
**

**Supplementary Figure 6: Binding of bevacizumab to HUVEC is concentration dependent.** HUVEC pre-seeded onto collagen coated 96-well plates were incubated with serial dilutions (2nM-405nM) of bevacizumab (top row) or aflibercept (bottom row) in the presence of 10nM VEGF_165_ at 37°C. Surface bound inhibitor complexed with VEGF_165_ was detected by incubation with A488 anti-hIgG (green fluorescence) at 4°C. Cells were washed, fixed with 4% paraformaldehyde and incubated with a nucleic acid counterstain (DRAQ5, red fluorescence) prior to analysis. The highest binding to HUVEC was observed at 15 nM bevacizumab or 10 nM aflibercept Scale bar = 100μm.

**A)**

**B)**

**Supplementary Figure 7. In vitro binding of bevacizumab and aflibercept to NRP1.mFc and Heparin-biotin by SPR (Biacore) in the presense of VEGF-A_165_.** (A) Human neuropilin-1 (NRP1.mFc) (155 RU) were captured on a anti-mouse Fc coupled chip surface. Sensograms show bevacizumab and aflibercept at a concentration of 5nM in a 1:1 ratio pre-complexed with or without human VEGF-A_165_. (B) Heparin-biotin (35 RU) were captured on a neutravidin coupled chip surface. Sensograms show bevacizumab and aflibercept at a concentration of 5nM in a 1:1 ratio pre-complexed with or without human VEGF-A_165_.
